# Transcriptional profile of ribosome-associated quality control components and their associated phenotypes in mammalian cells

**DOI:** 10.1038/s41598-023-50811-z

**Published:** 2024-01-16

**Authors:** Otávio Augusto Leitão Dos Santos, Rodolfo L. Carneiro, Rodrigo D. Requião, Marcelo Ribeiro-Alves, Tatiana Domitrovic, Fernando L. Palhano

**Affiliations:** 1 Programa de Biologia Estrutural, Instituto de Bioquímica Médica Leopoldo de Meis, Universidade Federal do Rio de Janeiro, Rio de Janeiro, RJ 21941-902 Brazil; 2 Departamento de Genética, Evolução, Microbiologia e Imunologia, Instituto de Biologia, Universidade Estadual de Campinas, Campinas, SP Brazil; 3 Fundação Oswaldo Cruz, Instituto Nacional de Infectologia Evandro Chagas, Rio de Janeiro, 21040-900 Brazil; 4 Departamento de Virologia, Instituto de Microbiologia Paulo de Góes, Universidade Federal do Rio de Janeiro, Rio de Janeiro, 21941-902 Brazil

**Keywords:** Computational biology and bioinformatics, Genome informatics

## Abstract

During protein synthesis, organisms detect translation defects that induce ribosome stalling and result in protein aggregation. The Ribosome-associated Quality Control (RQC) complex, comprising TCF25, LTN1, and NEMF, is responsible for identifying incomplete protein products from unproductive translation events, targeting them for degradation. Although RQC disruption causes adverse effects on vertebrate neurons, data regarding mRNA/protein expression and regulation across tissues are lacking. Employing high-throughput methods, we analyzed public datasets to explore RQC gene expression and phenotypes. Our findings revealed widespread expression of RQC components in human tissues; however, silencing of RQC yielded only mild negative effects on cell growth. Notably, TCF25 exhibited elevated mRNA levels that were not reflected in the protein content. We experimentally demonstrated that this disparity arose from post-translational protein degradation by the proteasome. Additionally, we observed that cellular aging marginally influenced RQC expression, leading to reduced mRNA levels in specific tissues. Our results suggest the necessity of RQC expression in all mammalian tissues. Nevertheless, when RQC falters, alternative mechanisms seem to compensate, ensuring cell survival under nonstress conditions.

## Introduction

Protein synthesis is a highly regulated cellular process necessary for maintaining cellular homeostasis^1^. However, translation is prone to different errors that can cause ribosome stalling followed by ribosome collisions and premature termination. Stalled ribosomes can be a source of aberrant misfolded and dysfunctional polypeptides, which can aggregate and disrupt cellular homeostasis. In addition, stalled ribosomes are unable to initiate new translation cycles, affecting the entire dynamics of protein synthesis^2^. It has been estimated that ribosome stalling occurs in 0.4% of translation events in *Escherichia coli*^3^ and in 2–5% of HEK293 cells^4^; therefore, cells must constantly deal with defective translation byproducts. Ribosome stalling occurs when the elongation process is interrupted by either the presence of a premature stop codon or the absence of a termination codon, which lead to mRNA nonsense-mediated decay and nonstop decay, respectively^5–9^. Additionally, the presence of strong structural elements in mRNA, rare codons or polylysine coding tracts can impair elongation, causing ribosome stalling and mRNA no-go decay^5,10–16^.

Data currently available in the literature indicate that the positions of stalled ribosomes on the mRNA are important in determining how these ribosomes will be detected and rescued^17^. Ribosomes stalled on mRNA collide with other elongating ribosomes, forming complexes with unique structural features, such as disomes, which are two ribosomes with their 40S subunits touching each other^4,18–20^. Collided disomes are recognized by the E3 ligase ZNF598 (Hel2 in yeast)^4,18^. ZNF598 recruits downstream effectors in a ubiquitylation-dependent manner^4,14,18,21–23^. One effector is the ASCC3 helicase (Slh1 in yeast), which utilizes ATP to separate the ribosomal subunits^22,24^. These mRNA degradation pathways lead to the recycling of the 40S ribosomal subunit but leave the 60S subunit with an incomplete nascent peptide occupying the exiting tunnel and a tRNA linked to the incomplete polypeptide^5,13^.

Initially identified in yeast^25^, the Ribosome-associated Quality Control (RQC) complex acts on ribosomes stalled during elongation. The RQC is responsible for the ubiquitination and extraction of the nascent incomplete peptide, which also leads to 60S subunit recycling. The RQC complex consists mainly of the proteins LTN1, NEMF, and TCF25, which interact with each other, and the 60S subunit is bound to uncompleted polypeptides^25,26^. In the RQC pathway, ribosomes that stall and collide during translation are first sensed by factors that eventually promote subunit dissociation, a process mediated mainly by ZNF598^21^. ZNF598 is an E3 ligase that recognizes the unique structural architecture at the collision interface and ubiquitinates the 40S subunit, which leads to subunit dissociation and permits the association of the RQC with the 60S subunit^4,18,21,22,27^. After ribosome dissociation, NEMF binds to the aberrant 60S subunit contacting the polypeptide-bound tRNA, a conformation that confers specificity to aberrant 60S and prevents 40S subunit reassociation^17^. Moreover, NEMF (Rqc2 in yeast) promotes the mRNA-independent synthesis of the C-terminal Alanine-Threonine (CAT) tail^28^. This CAT tail has two distinct functions: it is a stress signal due to its hydrophobic nature, and it assists in the ubiquitination of the nascent peptide through the exposure of lysine residues that may be enclosed inside the ribosomal exit tunnel^25,29^. Following NEMF binding to 60S, the ubiquitination of the nascent peptide is performed by the E3 ligase LTN1, a process that apparently depends on TCF25^30,31^. The molecular function of TCF25 is still not fully understood, but as previously shown by our group, the expression of its yeast homolog, Rqc1, is regulated post-translationally by LTN1, perhaps as a negative feedback mechanism to ensure that the RQC complex is not overactivated^16^. It is worth noting that TCF25 was originally described as a transcription factor, and it was recently implicated in the regulation of NFAT (nuclear factor of activated T cells) expression in cardiomyocytes and the activation of T cells during papillary renal cell carcinoma^32–35^.

Defects in the RQC complex have been associated with neurodegeneration and protein aggregation. Chu et al. demonstrated that homozygous LTN1 knockout mice exhibited age-dependent loss of locomotor ability. In addition, pathological analyses evidenced dystrophic neurites, gliosis, vacuolated mitochondria, and the accumulation of soluble hyperphosphorylated tau in these mice. Although LTN1 is widely expressed in all tissues, motor and sensory neurons and neuronal processes were the most affected^36^. Choe et al. showed that deletion of LTN1 in yeast causes stalled nascent peptides, forming detergent-resistant aggregates and inclusions. This aggregation process depended on CAT tail formation by Rqc2, while the double knockout ΔLTN1ΔRQC2 prevented the formation of these aggregates in yeast^37^.

Similarly, CAT tailing by NEMF in HeLa (in mammals alanine tails) cells can form aggregates when LTN1 is depleted^38^. The dysfunction of NEMF is also associated with neurological disorders. Martin et al. reported that mice with the NEMF mutations R86S and R487G presented many neuromuscular phenotypes. Moreover, R86S mice died prematurely, with a median lifespan of 20 days. Furthermore, it has been previously observed that humans with mutations in NEMF display intellectual disability, speech delay, neuropathy, and motor dysfunction with varying severities and progression^39^.

Recently, it was shown that aged *Saccharomyces cerevisiae* and *Caenorhabditis elegans* under chronological aging downregulated the mRNA species of some RQC components and have increased ribosome stalling and collisions at polybasic tracts^40^. It was proposed that RQC activity is saturated by an age-dependent increment in ribosome stalling events, which could lead to impaired proteostasis, as observed in older individuals. However, it was not demonstrated that aging affects the activity and expression of RQC components in mammalian tissues.

The above evidence indicates that neuronal cell physiology is particularly affected when the RQC complex is perturbed. It is unclear, however, whether the expression of the components of RQC varies across different tissues. Additionally, it is not known if the mRNA expression levels of the RQC components are co-regulated, and if aging perturbs the expression. We took advantage of publicly available results from high-throughput experiments such as transcriptome, proteome, and Perturb-Seq datasets (as listed in Table 1) to map various aspects of RQC component expression and regulation. Although ZNF598 is not regarded as an integral part of the RQC complex^4^, for the sake of simplicity, hereafter, it will be referred to as an RQC component.

**Table 1:** Public datasets assessed in the study.

Data	Source	Identifier
Proteome and transcriptome abundance	Wang et al.^51^	
Proteome and transcriptome abundance	Jiang et al.^52^	
mRNA half-life	Agarwal and Kelley^53^	
Translation efficiency	Chothani et al.^54^	
Protein half-life	Mathieson et al.^55^	
Dependency map	Tsherniak et al.^56^	https://depmap.org/portal/
Haploinsufficiency score	Collins et al.^57^	
Perturb-Seq CRISPRi gene silencing	Replogle et al.^58^	https://gwps.wi.mit.edu/
CRISPR‒Cas9-based screening for GFP fluorescence level	Hickey et al.^47^	
Transcriptomes of human organs and tissues in age clusters	The GTEx Consortium^59^	https://www.gtexportal.org
Transcriptomes of mouse organs and tissues in age clusters	The Tabula Muris Consortium et al.^60^	https://tabula-muris.ds.czbiohub.org/

We focused our analyses on the RQC complex (LTN1, TCF25, NEMF, and ZNF598), but we also extended some analyses to other factors related to the co-translational regulation of collided ribosomes (EDF1, GIGYF1, GIGYF2, EIF4E2, RACK1, ANKZF1, ASCC1, ASCC2, and ASCC3). It was recently demonstrated that EDF1 is a ZNF598-independent sensor of ribosome collisions. EDF1 recruits the translational repressors GIGYF2 and EIF4E2 (also called 4EHP) to collided ribosomes to initiate a response that prevents new ribosomes from translating defective mRNAs^41,42^. The GIGYF2–EIF4E2 complex competes with the translation factor eIF4E for binding the 5′ cap structure^43^. This allows a dynamic response locally and temporally, such that only if the collision persists would there be recruitment of the RQC complex via ZNF598^44^. Decreasing the translation initiation rates would also reduce the collision rate. Thus, ribosome collisions as an important parameter that cells use to adjust the quality and quantity of translational products^42^. Additionally, 4EHP–GIGYF1/2 complexes trigger co-translational mRNA decay^45^.

Moreover, EDF1 recruitment to collided ribosomes requires the presence of RACK1 (Asc1 in yeast), which is located at the interface of collided ribosomes^4,46,47^. RACK1 has been identified as a protein present in the small subunit of eukaryotic ribosomes. It acts as a scaffolding protein capable of interacting with numerous partners and recruiting translation regulators. RACK1 provides an interface for recognition by ZNF598, allowing the separation of ribosomal subunits and the downstream recruitment of RQC^48,49^. Additionally, the ASC-1 complex, formed by ASCC1, ASCC2, and ASCC3 (helicase), separates the ribosomal subunits in an ATP-, ZNF598-, and ubiquitin-dependent reaction^24,50^.

We found that all RQC and RQC-associated components are ubiquitously expressed in the analyzed tissues. Interestingly, TCF25 showed a high number of mRNA transcripts, which does not translate into high protein levels in most tissues analyzed. In addition, when compared to the rest of the genome, TCF25 had an increased mRNA half-life coupled with a shorter protein half-life, suggesting post-translational regulation. To test this experimentally, we evaluated TCF25 expression in HEK cells by western blot and observed that TCF25 protein levels can be increased by inhibiting the proteasome, indicating that post-translational regulation determines the TCF25 expression level. By analyzing published Perturb-Seq data, we observed that silencing RQC components caused only mild effects under normal growth conditions in different cell lines. Finally, we dissected the effects of aging on the mRNA levels of the RQC components in humans and mice. In general, aging caused a discrete decrease in mRNA levels in some human tissues. Moreover, we found that LTN1 and NEMF expression levels and associated phenotypes tended to be similar, while TCF25 and ZNF598 had specific, uncorrelated expression patterns. In conclusion, our results indicate that RQC components are ubiquitously expressed in mammalian tissues, and we found support for aging-dependent downregulation that might regulate RQC function. Additionally, although TCF25 interacts with LTN1 and NEMF, it is subjected to differential expression regulation.

## Materials and methods

### Human and animal data

No animal or human samples were used directly in this research. Data from human and animal models were obtained from published public datasets and articles, as shown in Table 1.

### Transcriptome and translatome profiles

To assess the transcription and translation profiles of the components of the RQC complex, we obtained data from Wang et al.^51^ obtained from 29 healthy human tissue samples. For each gene of interest, the transcripts per million (TPM) values were used to estimate the Z-score ((x – mean)/standard deviation) for each gene across the tissue, and we plotted the results as a heatmap. To study the proteome in each tissue, we used the dataset containing relative protein abundances (log_2_) and estimated the Z-score for each gene. A similar approach was performed with the Jiang et al.^52^ dataset in the GTEx Consortium. From these data, we obtained the transcriptomes and proteomes of 114 samples from 32 organs from 14 individuals, with replicates ranging from 1 to 22.

### RNA and protein half-lives

To assess the half-lives of transcripts for each gene in the RQC complex, we used the dataset produced by Agarwal and Kelley^53^. They generated a compendium of mammalian (mouse and human) mRNA half-life datasets from 33 publications obtained from cell culture and normalized the values using the Z-score. For protein half-life, we assessed the data from five different non-dividing cell types (human B cells, monocytes, NK cells, hepatocytes, and mouse embryonic neurons) by Mathieson et al.^55^. We calculated mean protein half-life values considering all cells and compared them with the values for each component of the RQC complex in each cell line analyzed.

### Translational efficiency

To analyze the mRNA translation efficiency (TE), we downloaded the dataset from ribosome profiling experiments published by Chothani et al.^54^. We then used the RNA-seq and Ribo-seq data for each gene in the cell lines utilized and calculated the TE with the following equation:

$${\text{X}}\, = \,{\text{Log}}_{{2}} \left( {{\text{Ribo-seq}}} \right)/{\text{Log}}_{{2}} \left( {{\text{RNA-seq}}} \right).$$

### Impact on growth analyses

The impact of the deletion of RQC complex component genes was obtained using large-scale gene essentiality data from the DepMap project^56^. The authors of the dataset generated a genome-scale library of 100,000 short hairpin RNAs (shRNAs) to analyze genome-scale loss-of-function screens performed in 1086 human cell lines. A scoring system was created to quantify the effects of gene inactivation on cellular growth in specific cell lineages. If the gene inactivation caused no effect in a specific cell lineage, a score equal to zero was attributed to this pair (cell line/gene inactivated). Conversely, a score of − 1 meant that this pair led to cell death, and it corresponded to the median of all common essential genes. Data were obtained directly from the DepMap portal (https://depmap.org). We searched in “perturbation effects” and downloaded the dataset (DepMap 23Q2 Public + Score, Chronos) for each RQC complex gene. Furthermore, we plotted the correlations between growth score values obtained for NEMF and LTN1 silencing in different cell types.

### Haploinsufficiency score

The haploinsufficiency score was created through the meta-analysis of approximately one million individuals in both disease and control cohorts to identify disease-associated dosage-sensitive regions, allowing for dosage sensitivity for each gene and creating a haploinsufficiency score^57^. We used the score obtained for each gene of interest for comparison purposes. Genes with values equal to or greater than 0.86 were considered haploinsufficient.

### Perturb-Seq CRISPRi gene silencing

To evaluate the effects of RQC impairment at the transcriptional level, we analyzed data from Replogle et al.^58^, in which genome-scale disturb-seq was performed in the leukemia K562 cell line, and investigated the mRNA expression profiles of individually silenced genes. The data were obtained from the online platform https://gwps.wi.mit.edu/, which separates the top 30 up- and downregulated genes for each selected gene. We used the K562 Genome-Wide Perturb-Seq database, selected the top 30 up- and downregulated component genes of the RQC complex, and separated them into clusters to understand the transcriptional profile. Additionally, we used the 10 genes with the most similar transcriptional profiles provided by the tool for each RQC complex component.

### Gene ontology

We selected gene clusters (up- and downregulated) that presented similar transcriptional profiles between NEMF and LTN1 and performed a gene ontology analysis. The gene set enrichment ontology analyses were performed in the Gene Ontology Consortium (http://geneontology.org/) with the GO Enrichment Analysis tool, searching for “Biological process”. We selected the results with the highest fold enrichment.

### FACS-based CRISPRi screen

To determine if the candidate genes participate in the RQC complex, we used data from Hickey et al.^47^, which measures the expression levels of fluorescent reporters lacking stop codons (leading to nonstop decay) in a genome-wide CRISPRi screening. Positive values indicate that the knockdown of that gene stabilizes the GFP signal. We used the RQC complex component as a control. We used the average calculated from the two replicates.

### Age-dependent transcriptome

To assess whether the transcriptional profile of RQC components changes with age, we used data from the GTEx consortium for human tissues^59^ and The Tabula Muris Consortium for mouse tissues^60^. We downloaded the data from the GTEX consortium data from the website https://www.gtexportal.org on September 2022 for each human tissue and separated them into age clusters (20–29, 30–39, 40–49, 50–59, 60–69, and 70–79). We used the transcripts per million (TPM) values to determine the mRNA abundance across the human tissues in different age clusters. The data were normalized according to the reference age range (20–29). For mouse data, we used The Tabula Muris Consortium, which offers transcriptomes of 20 organs from four male and three female mice ranging from 1 to 27 weeks old. The data handling and presentation were similar to those of human samples.

### Proteasome and autophagy inhibition

For the proteasome inhibition and autophagy assay, we used 1 × 10^6^ HEK 293 cells/well cultured in a 6-well plate. After 24 h, the medium was removed, and the cells were gently washed with 1× PBS pH 7.4 and incubated with 10 µM MG-132 (proteasome inhibitor) and 50 µM chloroquine (autophagy inhibitor) at 37 °C for 3 h in the presence of 100 µg/ml cycloheximide. After this period, the culture medium was removed, 100 μl of ice-cold RIPA (Thermo Fisher) lysis buffer with 1 mM PMSF and 1× Halt™ Protease and Phosphatase Inhibitor Cocktail (Thermo Fisher) was added, and the cells were scraped. All these procedures were performed on ice. Cells were added to microtubes, lysed with a microtube macerator, and centrifuged at 9000 rpm for 15 min at 4 °C. Then, the supernatants were collected, 4× sample buffer was added, and the samples were heated at 100 °C for 10 min to perform Western blotting.

### Western blotting

Proteins separated by 12.5% SDS‒PAGE were transferred to PVDF membranes using a Bio-Rad TRANS-BLOT semidry system in transfer buffer (25 mM Tris, 192 mM glycine, 0.1% SDS, 20% methanol, pH 8.3). Membranes were blocked using LI-COR blocking buffer for 2 h at 4 °C. Incubation with primary antibodies was performed at 4 °C overnight. Membranes were incubated in LI-COR Odyssey secondary antibodies for at least 3 h and then observed in an Odyssey scanner. The primary antibodies used were anti-Nulp1 (TCF25) mouse (Invitrogen; 1:3000 dilution) and anti-β-actin mouse (Thermo Scientific; 1:5000 dilution). The secondary antibody was anti-mouse 800 CW LI-COR (1:5000 dilution).

## Results and discussion

### RQC mRNA and protein expression across human tissues

**Figure 1:**
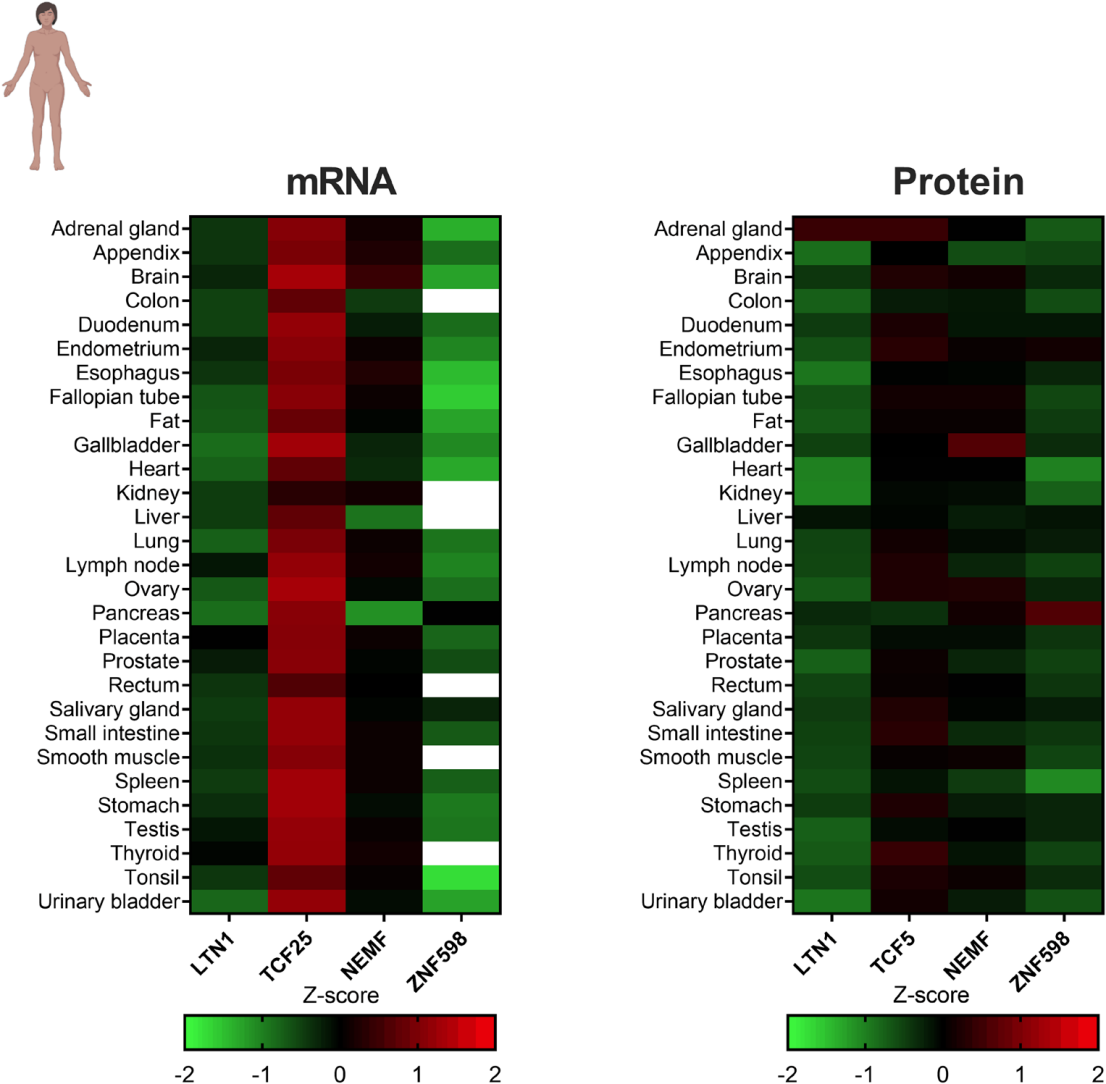
Heatmap of the Z-scores distributions of the RQC components calculated from the transcriptomes and proteomes of human tissue samples. Data obtained from Wang et al.^51^. White rectangles denote the absence of data. Each rectangle represents the expression of one of the RQC genes against the average expression of the whole transcriptome/proteome in a particular tissue. The heatmap analyses compare the expression of a single transcript (e.g., TCF25) against the average expression of the whole transcriptome in a particular tissue. The mRNA expressions of TCF25 in most samples are red colored because the mRNA expression of TCF25 is higher than the average total genome mRNA expression in the analyzed tissues.

To describe and compare the expression levels of the RQC components in different human tissues, we curated previously published work that generated paired transcriptome and proteome datasets of human samples. Wang et al.^51^ investigated the transcriptome and proteome from 29 different healthy human tissues from the Human Protein Atlas project. Using this dataset, we investigated the mRNA and protein expression levels of LTN1, TCF25, NEMF, and ZNF598 (Fig. 1). Transcripts were compared by determining the Z-score for each gene using the transcripts per million (TPM) values. Protein levels derived from proteome data were compared by determining the relative protein abundance (log_2_) (Fig. 1). All RQC components were expressed in most tissues analyzed at roughly similar levels. Therefore, even though RQC malfunction particularly impacts the nervous system in vertebrates^36,39^, this specificity could not be explained by the expression levels of RQC components. LTN1 and ZNF598 showed lower mRNA expression levels, while NEMF showed a Z-score close to zero for each tissue. Surprisingly, TCF25 showed mRNA expression levels that were approximately twofold higher than the average of gene expression levels in most tissues. The protein and mRNA expression levels showed similar patterns for LTN1, NEMF, and ZNF598, with the exception of TCF25. As mentioned above, the mRNA expression levels for TCF25 were higher than the average gene expression in every tissue analyzed, and it was the most abundant RQC component transcript (Fig. 1). However, this high mRNA expression level was not reflected in protein levels, which were generally similar to those of the other RQC components.

In their study, Wang et al.^51^ used only one sample per tissue to generate the protein and mRNA expression data. Thus, the small sample size undermined the formation of a solid conclusion about subtle expression variations between tissues. However, despite this limitation, this dataset allowed us to observe that (1) both the mRNAs and proteins of the RQC components are expressed in most tissues and (2) TCF25 mRNA is expressed at high levels, while its protein is expressed at levels closer to the average in most tissues (Fig. 1).

**Figure 2:**
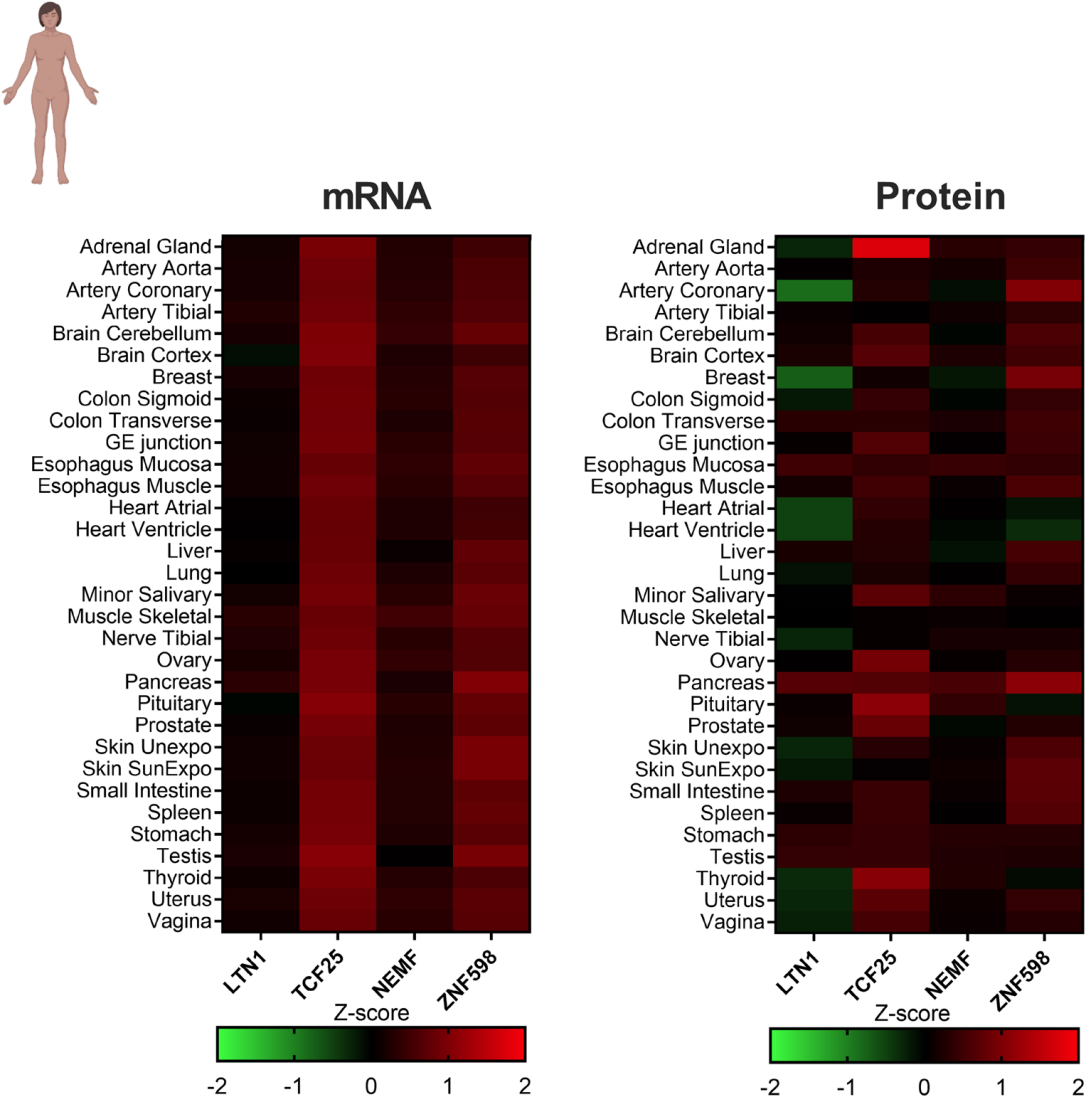
Heatmap of the Z-Scores distributions of the RQC components calculated from the transcriptome and proteome of 114 samples of 32 major organs from 14 different individuals, with replicates ranging from 1 to 22. Data obtained from Jiang et al.^52^.

We repeated the same analysis using another dataset with a larger sample size. Jiang et al.^52^ analyzed the transcriptome and proteome of 114 samples from 32 major organs from 14 different individuals, with biological replicates ranging from 1 to 22. As illustrated in Fig. 1, we used Z-score values to compare the mRNA and protein expression levels of the RQC components (Fig. 2). We observed that, overall, the expression levels were similar to or higher than the average of the genome (Fig. 2). For NEMF and ZNF598, the protein expression levels were slightly higher than the global average, showing positive correlation with their mRNA expression levels. Moreover, the TCF25 mRNA expression levels were above the 75th percentile (3rd quartile) in all 32 tissues studied. However, the TCF25 protein expression levels were above the 75th percentile in only 10 out of 32 tissues, namely, the adrenal gland, brain cortex, minor salivary, ovary, pancreas, pituitary, prostate, thyroid, uterus, and vagina. For the other 22 tissues, the protein Z-scores values for TCF25 were relatively lower than the mRNA Z-score (Fig. 2).

The two datasets indicated that the TCF25 protein expression levels were relatively low compared to the mRNA expression levels detected in many tissues compared to other RQC components (Figs. 1 and 2). This observation suggests that TCF25 may be regulated at the level of translation and/or post-translation.

When we looked into the regulation of the RQC-associated genes, we observed similar mRNA and protein expression levels, considering the data from Wang et al.^51^. It is interesting to highlight that the mRNA and protein expression levels were significantly high for EDF1 and RACK1 (Fig. S1). When evaluating the data from Jiang et al.^52^, we observed high levels of RACK1 mRNA that did not correlate with its protein expression (Fig. S2). RNA transcripts are determinants for protein abundance, and in most cases, higher mRNA levels lead to higher protein abundance^61^. However, many factors can affect the mRNA/protein ratio in a dynamic and complex process^62^. The amount of protein generated by mRNA can result from different, non-mutually exclusive factors or regulatory processes such as codon usage, mRNA half-life, and protein turnover^61,63^. As TCF25 was the only gene to display high mRNA levels and low protein levels in both datasets, we focused on the investigation of its regulatory processes.

**Figure 3:**
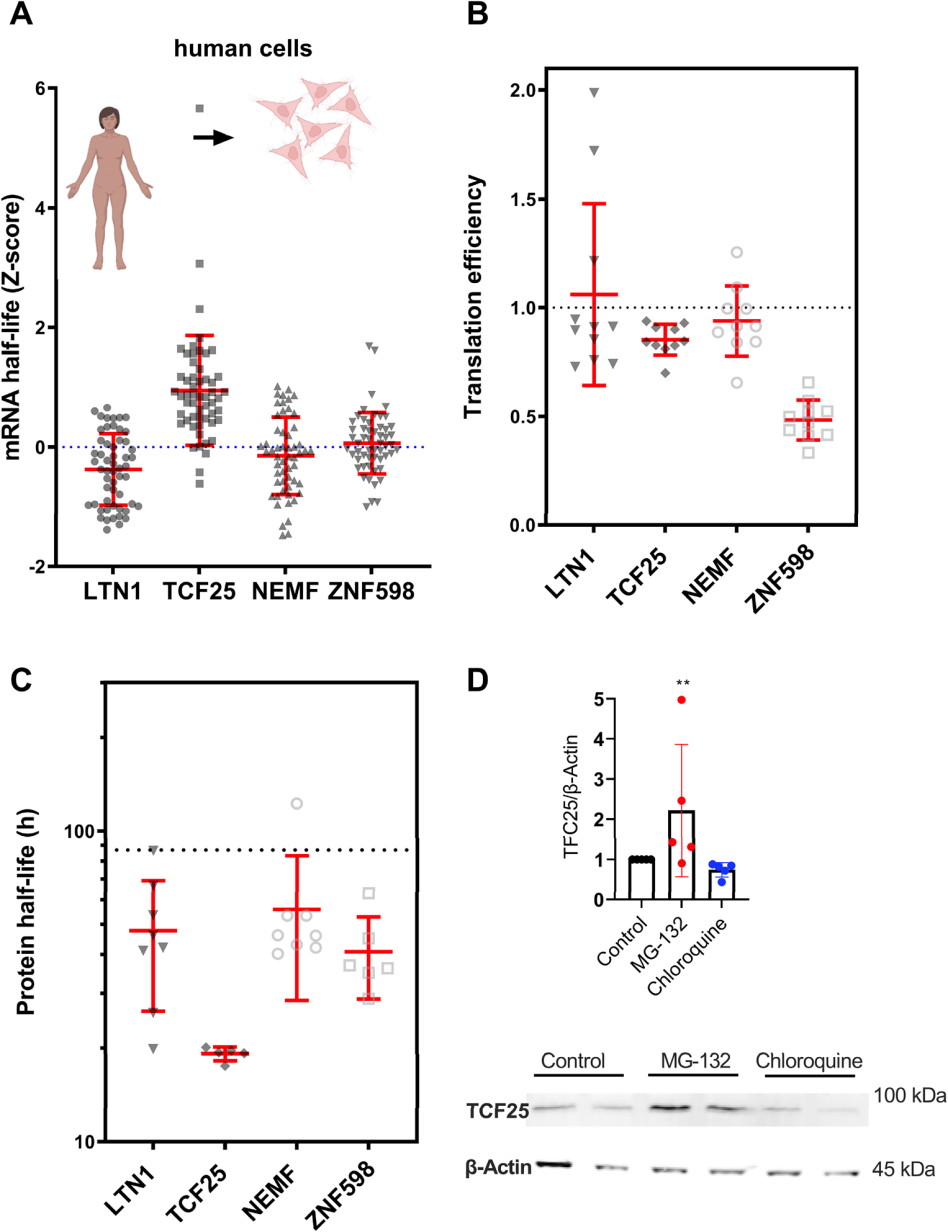
Factors that affect the abundance of RQC complex components. (**A**) Z-scores calculated for the mRNA half-lives of RQC component transcripts in human cells. Data obtained from Agarwal and Kelley^53^. (**B**) Log_2_ values of the mRNA translation efficiency of each transcript from six primary human cell types and five tissues. Data obtained from Chothani et al.^54^. (**C**) Protein half-lives for each RQC component. The dotted line refers to the average half-life for all proteins considering all cell lines tested (86.95 h). Data obtained from Mathieson et al.^55^. (**D**) Western blot of TCF25 from HEK293 cells treated for 3 h with 100 μg/ml cycloheximide to block protein synthesis. MG-132 (10 μM) or chloroquine (50 μM) was added to block proteasome or autophagy. DMSO was the control vehicle, and β-actin was measured as a loading control. ** p = 0.0085 by Friedman’s test.

To assess whether the relatively low TCF25 protein levels noted in the two datasets and illustrated in Figs. 1 and 2 could be related to processes involving mRNA stability, we evaluated the mRNA half-life of each RQC component (Fig. 3A). We used data produced by^53^, which generated a compendium of mammalian (mouse and human) mRNA half-life datasets from 33 publications obtained from cell culture. For human cells, the mRNA half-lives for LTN1, NEMF, and ZNF598 tended to be close to those of most transcripts in these cells (Fig. 3A, dotted line). In contrast, TCF25 showed a slightly higher mRNA half-life, with mean Z-score values of approximately 0.9. These findings indicate that the decrease in TCF25 protein expression level is not due to the short half-life of its mRNA. It is important to note that while the mRNA and protein quantifications presented herein were obtained from studies using human tissues (Figs. 1 and 2), the mRNA half-life data were obtained using cultured cells (Fig. 3A).

Another factor that can modulate the protein amount produced by mRNA is translation efficiency (TE). To investigate the effect of TE on TCF25 expression, we used a dataset from six primary human cell types and five tissues published by Chothani et al.^54^. They determined the translation elongation rates for the entire transcriptome through ribosome profiling. The TE values revealed that TCF25 accompanied NEMF and LTN1 (Fig. 3B); therefore, the translation efficiency was probably not the factor behind the relatively low TCF25 protein levels. We then asked if the tissues used to determine the TE of ZNF598 (liver, kidney, fat, heart, and brain tissues) presented lower protein levels (Figs. 1 and 2). However, no clear correlation was noted between the low TE values and the low protein/mRNA ratios observed for the aforementioned tissues.

We investigated protein half-lives and turnover using data from five different non-dividing cell types (human B cells, monocytes, NK cells, hepatocytes, and mouse embryonic neurons) by Mathieson et al.^55^. All RQC components showed shorter half-lives than the average, with TCF25 showing the lowest half-life of less than 20 h for all cell types tested (Fig. 3C). Analyses of the TE values and protein and mRNA half-lives of the RQC-associated proteins revealed similar patterns to the RQC proteins (Fig. S3).

As the transcriptome and proteome analyses suggested that the TCF25 protein has a fast turnover, we decided to determine whether TCF25 levels are controlled by any of the two main pathways for protein degradation: the proteasome and autophagic vesicles. To explore this question, HEK293 cells were treated with cycloheximide to block protein synthesis, and MG132 or chloroquine was added to inhibit the proteasome or autophagy, respectively. After a 3-h treatment (Fig. 3D), we measured the TCF25 levels by western blot. We detected an increase in TCF25 expression after proteasome inhibition when compared with the vehicle control. However, no difference was observed when autophagy was inhibited (Fig. 3D). We concluded that, at least for HEK293 cells, TCF25 protein expression levels are regulated by the proteasome.

### Effects of RQC component disruption on tissue cell survival and gene expression profiling

We observed that the components of the RQC complex are ubiquitously expressed in mammals, indicating their housekeeping role. In addition to the well-established co-translational quality control function described above, RQC components can be implicated in other processes, as in the case of TCF25. Nevertheless, gene deletion of RQC components revealed mixed results depending on the organism. Deletion of LTN1 has no detectable effect in yeast cells growing in optimal conditions, and cells with double knockout of LTN1 and Rqc1 are also viable^16,25^. These observations suggest that activation of the RQC complex may be necessary only for specific stress situations. Moreover, LTN1 knockout mice exhibit a strong neuromotor phenotype, and NEMF mutations in humans and mice also lead to neuromotor impairment^36,39^, indicating that RQC is necessary during neuronal development.

**Figure 4:**
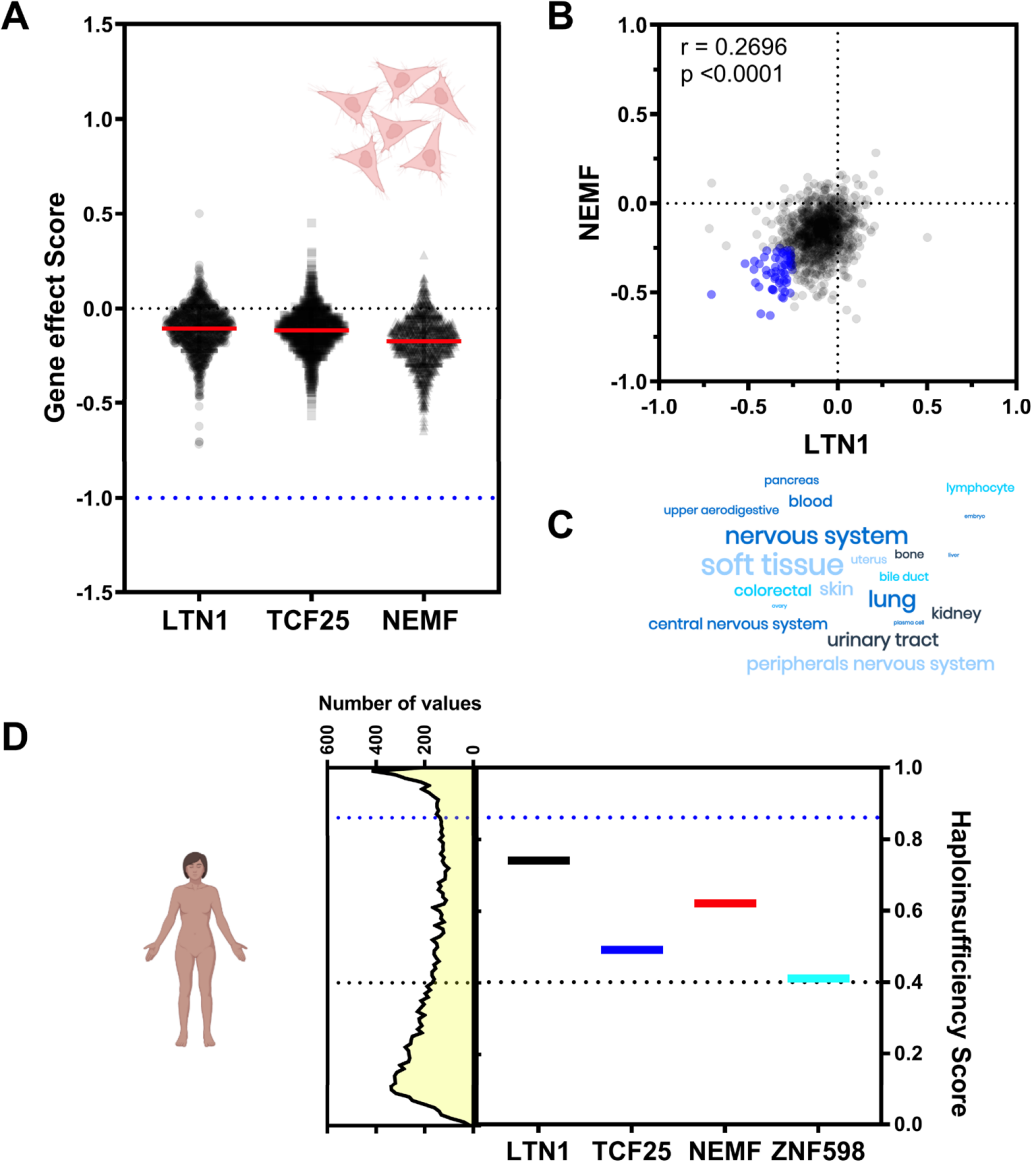
The genes encoding the components of the RQC complex are not essential. (**A**) Scores attributed to the impact of gene inactivation on the growth rate in 1086 cancer cell lines for LTN1, NEMF, and TCF25. Data obtained from Tsherniak et al.^56^. (**B**) Correlation graph of the silencing effect between NEMF and LTN1. (**C**) Word cloud representation of the cellular lineages more affected by NEMF and LTN1 silencing. (**D**) Haploinsufficiency scores for each component of the RQC complex and ZNF598. Genes with values equal to or greater than 0.86 are considered haploinsufficient. Data obtained from Collins et al.^57^.

To further examine to what extent RQC genes are fundamental components of living cells, we utilized large-scale gene essentiality data from the DepMap project^56^. A scoring system was created to quantify the effects of gene inactivation on cellular growth in a specific cell lineage^56^. If the gene inactivation caused no effect on cellular growth in a specific cell lineage, a score equal to zero (dotted black line, Fig. 4A) was attributed to this pair (cell line/gene inactivated). Conversely, a score of − 1 (blue dotted line, Fig. 4A) meant that this pair led to cell death and corresponded to the median of all common essential genes. The lowest score for the RQC components was − 0.7, meaning that they were not essential for the in vitro growth of mammalian cells (Fig. 4A). However, the median score for all RQC components analyzed was below zero, suggesting that the perturbation of the RQC causes some negative effect on cellular growth (Fig. 4A). Similar results were observed for RQC-associated components, except for RACK1 (Fig. S4A), which presented an average value of -1.85, demonstrating the importance of this gene for cell viability. The RACK1 protein has several functions in the cell and plays an important role in the separation of collided ribosomes. This importance for cell viability can be observed through its homolog in yeast Asc1, as knockout of this gene significantly affects yeast growth, with substantial frameshifting during translation^64^.

Another feature extracted from this dataset is the correlation between growth score values obtained with different silenced genes on different cell types. We found a positive correlation between NEMF and LTN1, meaning that the silencing of these genes causes a similar effect on growth depending on the cellular lineage analyzed (Fig. 4B). To determine which cellular lineages are more affected by NEMF and LTN1 silencing, we arbitrarily defined cutoffs of − 0.25 (blue circles in Fig. 4B) for both genes. Soft tissue and nervous system cellular lineages were the most affected by NEMF and LTN1 silencing (Figs. 4C and S5), which agrees with the neurological impairments observed in vivo^36,39^. The same correlation analyses described in Fig. 4B were performed for all RQC-related genes, and the Pearson correlation values were used to build a correlation matrix (Fig. S6). GIGYF2 and EIF4E2 demonstrated the highest positive correlation, followed by NEMF and LTN1. Finally, the ASCC complex components (ASCC1, ASCC2, and ASCC3) formed another cluster, highlighting the functional relationship of these proteins (Fig. S6). These data suggest that perturbing the core RQC components (NEMF/LTN1) or the RQC-associated proteins (ASCC complex and GIGYF2/EIF4E2) produces different effects that disturb specific cell types to different extents.

Another important consideration is to assess the haploinsufficiency of the RQC genes. Haploinsufficiency is defined as a dominant gene phenotype in diploid organisms, in which a single copy of the wild-type allele at a locus in a heterozygous combination with a variant allele is insufficient to produce the wild-type phenotype. In this context, we evaluated whether defects in one of the alleles of the genes of the RQC complex would be sufficient to alter the wild-type phenotype and thus correlate with the growth impairment observed in the knockout cells (Fig. 4C). In addition, a meta-analysis of approximately one million human individuals in disease and control cohorts was recently performed to identify disease-associated dosage-sensitive regions^57^. The authors predicted dosage sensitivity for each gene, creating a haploinsufficiency score. In other words, a score value equal to or higher than 0.86 (blue dotted line, Fig. 4D) meant that a single copy of the wild-type allele at a locus in a heterozygous combination with a variant allele was insufficient to produce the wild-type phenotype. When we analyzed the haploinsufficiency scores of the RQC components, we observed that all genes had scores lower than 0.86, meaning that these genes are not haploinsufficient because just a single copy of the wild-type allele is enough to produce the wild-type phenotype (Fig. 4D). These data agree with the description of families with pathogenic biallelic NEMF variants with a spectrum of central neurological disorders^39^. In conclusion, in vertebrate cellular lineages, knockdown of the RQC components causes a mild phenotype that can be exacerbated in specific tissues (Fig. 4A). These results suggest that even though the RQC components are ubiquitously expressed genes (Figs. 1 and 2), the cell can tolerate the translational perturbations caused by RQC impairment and continue growing, perhaps by adapting their transcriptional program. It is important to note that this scenario might be different under stress conditions. Furthermore, RACK1, GIGYF2, and EIF4E2 presented values close to 1, demonstrating that a single correct copy of these genes cannot maintain the wild-type phenotype (Fig. S4B). These results and the others explained above indicate that RACK1 is fundamental for correct cellular functioning.

### Effects of RQC component disruption on the tissue cell transcriptome

**Figure 5:**
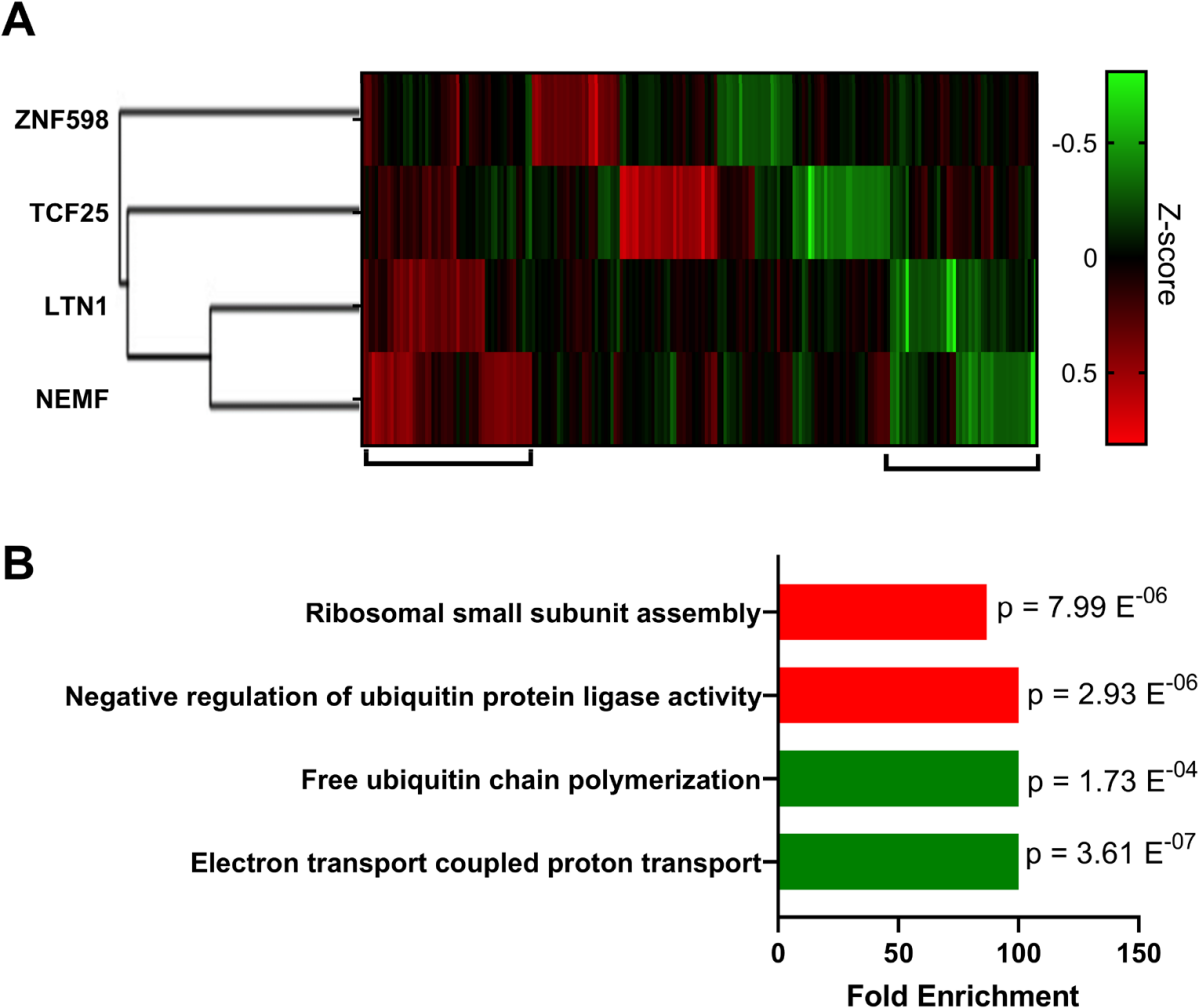
Transcriptional regulation elicited by RQC knockdown in human cells. (**A**) Heatmap of the Z-score distribution of the transcriptional profile when RQC genes were downregulated by CRISPR interference (CRISPRi) in the leukemia K562 cell line. Data obtained from Replogle et al.^58^. (**B**) Ontology enrichment of genes that showed the greatest similarity in their expression profiles when we silenced LTN1 and NEMF, as shown in Panel A. The results for up- and downregulated gene clusters are shown in red and green, respectively.

To understand how cells respond to RQC impairment at the transcriptional level, we analyzed data from Replogle et al.^58^, which performed genome-scale CRISPRi disturb-seq in the leukemia K562 cell line to determine the mRNA expression profiles of individually knockdown genes. The transcriptional responses were compared with those of the control cells. Using these data, we separated the top 30 up- and downregulated genes of the RQC components into clusters (Fig. 5A). As expected, LTN1 and NEMF showed similarities in both up- and downregulated genes, but the silencing of TCF25 showed different patterns (Fig. 5A). These results further support that TCF25 has additional functions other than co-translational control, such as the transcriptional regulation described above, and additional studies are necessary to evaluate which genes are regulated directly by this protein. We also performed an ontology enrichment analysis of the gene clusters that showed the greatest similarity in expression profile when we silenced LTN1 and NEMF. The enriched ontologies of the upregulated gene clusters were involved in negative regulation of the ubiquitination process and ribosome assembly (Fig. 5B). In contrast, the enriched ontologies for the downregulated gene clusters were involved in the electron transport chain and free ubiquitin chain polymerization (Fig. 5B).

**Figure 6:**
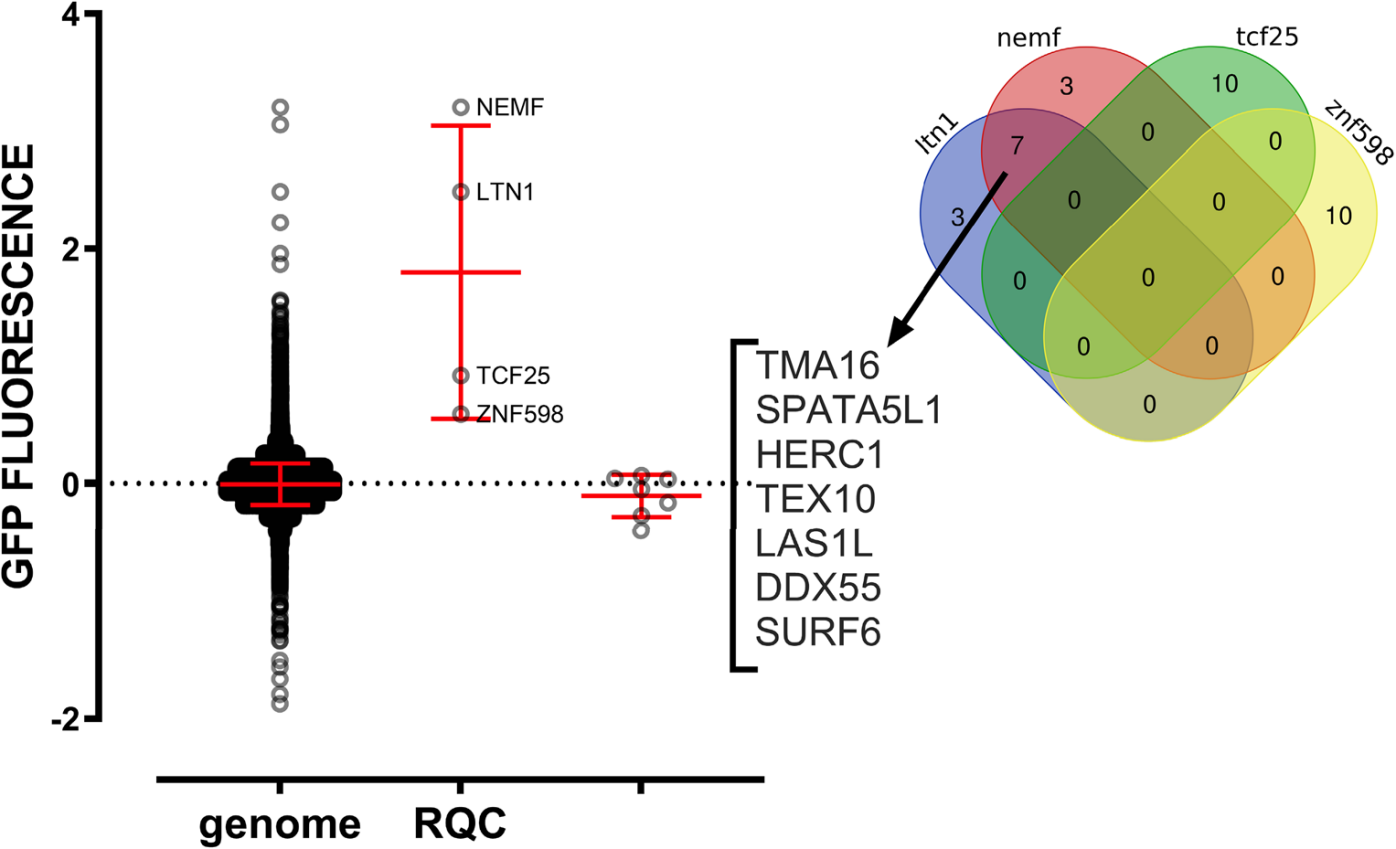
CRISPRi knockdowns of genes that produced similar transcriptional phenotypes to LTN1 and NEMF were not directly involved in RQC function (Right). Venn diagram showing the top 10 knockdown genes with similar transcriptional responses for each RQC component and ZNF598, of which 7 genes have a similar profile to NEMF and LTN1 knockdown. (Left) Graph of GFP fluorescence showing increased fluorescence levels when the components of the RQC complex are deleted, which was not observed for any of the 7 genes with similar transcriptional profiles. Data obtained from^47^.

Another interesting feature extracted from the Perturb-Seq data is a list of silenced genes presenting similar transcriptional responses. Figure 6 shows a Venn diagram that compares the top 10 genes that generated the most similar transcriptional phenotype to knockdowns of the RQC components. None of the 4 RQC genes produced similar transcriptional responses (top 10). Moreover, the ZNF598 and TCF25 lists showed no overlap whatsoever, while LTN1 and NEMF had 7 hits in common (TMA16, SPATA5L1, HERC1, TEX10, LAS1L, DDX55, and SURF6). Interestingly, none of these 7 genes were previously described in the RQC pathway and have diverse functions with no obvious connections with ribosome quality control or proteostasis (Supplementary Table 1). To check whether these genes could participate in co-translational quality control, we used data from Hickey et al.^47^, which measured the expression of fluorescence reporters lacking stop codons (leading to nonstop decay) in a genome-wide CRISPRi screening. As expected, the knockdown of LTN1 and NEMF greatly enhanced GFP fluorescence, while knockdown of TCF25 and ZNF598 showed smaller but statistically significant results (Fig. 6). In contrast, knockdown of none of the 7 genes identified in Perturb-Seq analyses (Fig. 6) affected the GFP fluorescence level, indicating that they are not directly involved in co-translational quality control pathways (Fig. 6). As expected, knockdown of GIGYF2, EIF4E2, RACK1, and ASCC3 showed high levels of GFP fluorescence (Fig. S7), as they are proteins involved in the identification and separation of collided ribosomes. Deleting ASCC1 and ASCC2 individually did not significantly affect the functioning of the RQC complex. However, a double knockout of these genes may have significant effects. Experiments with homologs in yeast (RQT complex) demonstrated that Slh1 (the yeast homolog of ASCC3) was fundamental for the complex. At the same time, the other components of the RQT only acted as cofactors facilitating Slh1 function^65^.

### Effects of aging on the mRNA levels of the RQC components

Proteostasis failure with the accumulation of insoluble aggregates is a hallmark of several neurodegenerative diseases^66,67^. Failure of the RQC, more specifically of LTN1, can lead to the accumulation of CATylated peptides that promote the formation of protein aggregates due to the hydrophobic nature of the CAT tail. These aggregates can disrupt neuronal morphogenesis^26,38,68^. Using yeast as a model, it was shown that aging causes saturation of the RQC complex, leading to the accumulation of insoluble aggregates of a reporter that is a target of the RQC^40^. Moreover, in yeast and worms, the mRNA expression levels of some RQC components decreased during aging. To assess whether the mRNA expression levels of the RQC components are affected by aging in mammals, we evaluated the transcriptomes from several tissues from donors with different age ranges obtained from the GTEx consortium.

**Figure 7:**
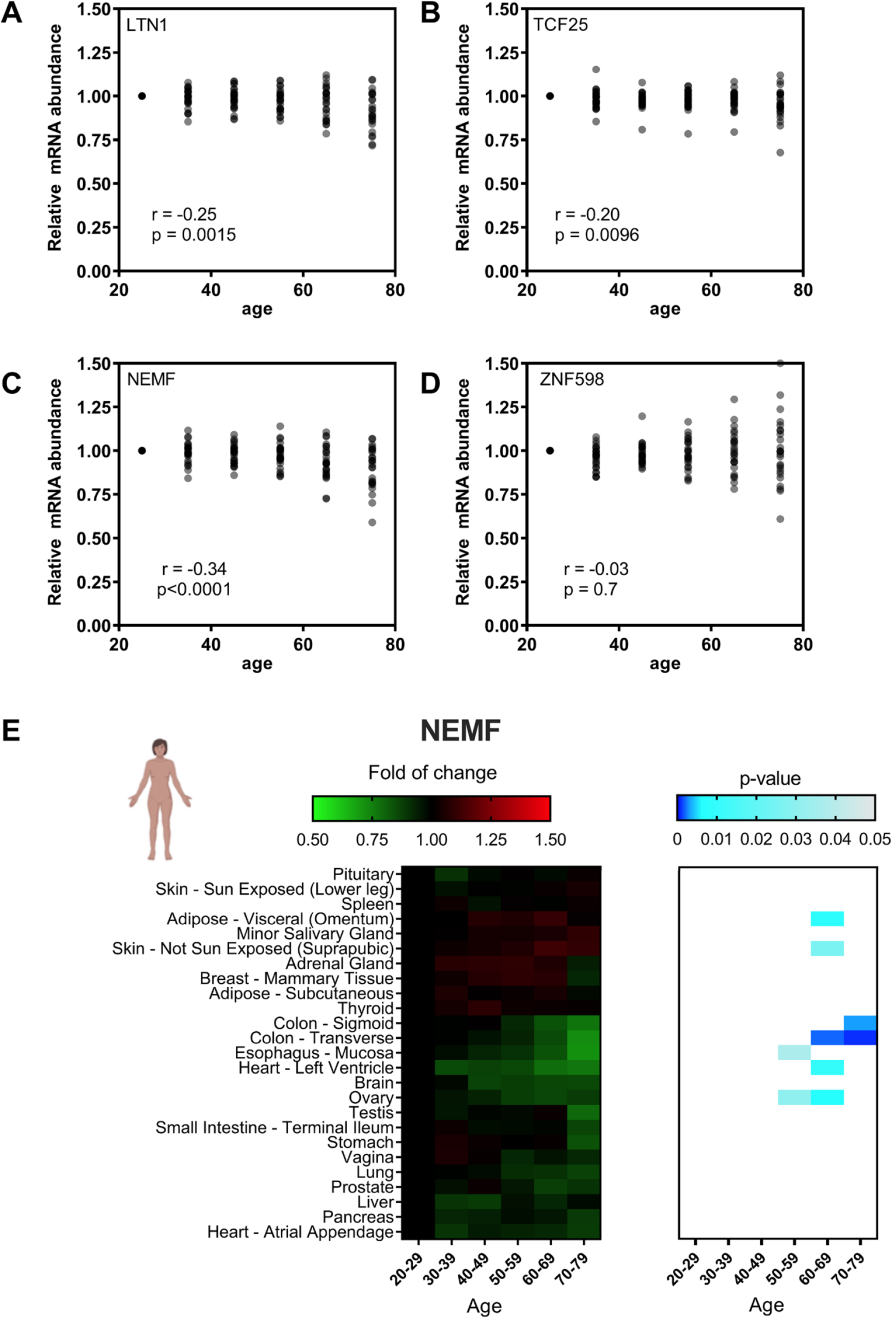
Effects of cellular aging on the transcriptional profiles of NEMF, LTN1, TCF25, and ZNF598 in different human tissues. (**A**–**D**) Comparison of the relative mRNA abundance levels in human tissues over various age ranges (20–80 years). Data obtained from the GTEx consortium. (**E**) Distribution of the Z-scores of the relative abundance of NEMF mRNA in human organs in different age clusters. Statistical significance was calculated using the p-value and is presented in a blue gradient for those relevant samples. All data were normalized to the reference age range (20–29).

We used the transcripts per million (TPM) values to determine the mRNA abundances across the human tissues at different ages (Fig. 7A–D). Compared with the youngest individuals, the mRNA expression levels of the RQC components in aged tissues showed a slight downward trend, which was more evident for NEMF, followed by LTN1 (Fig. 7A–D). TCF25 also showed a statistically significant decrease (Fig. 7B and C), while no difference was observed for ZNF598 (Fig. 7D). We then performed a cluster analysis to analyze tissue-specific age-dependent regulation. NEMF and LTN1 showed similar patterns of transcriptional regulation. Both genes showed repression in 5 tissues with significant p-values (Figs. 7E and S8). TCF25 and ZNF598 showed a more complex and diverse patterns of regulation (Figs. S9 and S10). While there was no statistically significant difference in the brain, mean reduction trends were observed for TCF25, LTN1, and ZNF598 mRNA expression.

We sought another model to test the age-dependent regulation observed with GTEx data. We repeated the analyses described in Fig. 7 using data from the *tabula muris* project^60^, which provided transcriptomes from 20 organs from four male and three female mice ranging from 1 to 27 weeks old. No age-dependent regulation was observed for the RQC components (Figs. S11–14), probably due to the limited number of samples available.

## Conclusion

Our results suggest that the RQC complex components are widely expressed in human tissues, suggesting that the RQC is part of the housekeeping processes of the cell (Figs. 1 and 2). LTN1 and NEMF share similarities in their expression levels (Figs. 1 and 2) and their functional and transcriptional phenotypes. However, TCF25, the third essential component of the RQC complex, showed a distinct regulation profile that produced different phenotypes compared with LTN1 and NEMF. This observation can be explained by functions of TCF25 outside of the RQC^32–35^.

Our results also showed that TCF25 had a high mRNA expression level, a low protein expression level, a high mRNA half-life, and a low protein half-life (Fig. 3). We detected an increase in TCF25 protein levels after blocking the proteasome (Fig. 3D). These results support a post-translational regulation of TCF25 expression, which has already been experimentally examined in the literature in yeast cells^16^. It has been proposed that the RQC complex co-translationally regulates Rqc1/TCF25 expression co-translationally through the ubiquitination of its polybasic region promoted by RQC activity^25^. However, our group has recently shown that this regulation occurs post-translationally and through the activity of LTN1 alone^16^. TCF25 regulation may impact RQC complex activity or the transcriptional control exerted by TCF25 in the nucleus. Further studies will be necessary to understand how these functions are coordinated and if there are tissue specificities for the molecular roles of TCF25.

Furthermore, we observed significant differences in the mRNA expression levels of the RQC components when we compared human tissues from young and aged individuals. Similar effects were observed with *S. cerevisiae* and *C. elegans*^40^. NEMF and LTN1 showed the most consistent age-dependent downregulation pattern in humans. In *C. elegans*, NEMF and TCF25 homologs showed repression in aged specimens, while in yeast, LTN1, NEMF, and ZNF598 homologs were affected^40^.

We observed a trend of decreased mean expression of the RQC components in the human brain (although not statistically significant), which could be relevant to age-related neuronal impairment (Figs. 7E, S2 and S3); in contrast, ZNF598 showed a trend toward increased expression, perhaps as a response to the failure of the RQC (Fig. S4). These findings provide insights into the regulation of the components of the RQC complex and its tissue-specific patterns, which could help clarify some phenotypes observed when the activity of the complex is affected by mutations or during aging. The limitations of our study included the comparisons between whole-genome data and tissue data, as well as cell data from mouse and human cells. However, we hope that some insights presented herein might be further tested experimentally.

### Supplementary Information

Supplementary Information.

## Data Availability

The data presented in this study were obtained from public datasets, as explained in Table 1. All western blotting images are shown in the supplementary information. More details about the datasets used and analyzed during the current study are available from the corresponding author upon reasonable request.

## References

[1] Yip MCJ, Shao S (2021). Detecting and rescuing stalled ribosomes. Trends Biochem. Sci..

[2] Howard CJ, Frost A (2021). Ribosome-associated quality control and CAT tailing. Crit. Rev. Biochem. Mol. Biol..

[3] Moore SD, Sauer RT (2007). The tmRNA system for translational surveillance and ribosome rescue. Annu. Rev. Biochem..

[4] Juszkiewicz S (2018). ZNF598 is a quality control sensor of collided ribosomes. Mol. Cell.

[5] Lykke-Andersen J, Bennett EJ (2014). Protecting the proteome: Eukaryotic cotranslational quality control pathways. J. Cell Biol..

[6] Losson R, Lacroute F (1979). Interference of nonsense mutations with eukaryotic messenger RNA stability. Proc. Natl. Acad. Sci. USA.

[7] Maquat LE, Kinniburgh AJ, Rachmilewitz EA, Ross J (1981). Unstable beta-globin mRNA in mRNA-deficient beta o thalassemia. Cell.

[8] Van Hoof A, Frischmeyer PA, Dietz HC, Parker R (2002). Exosome-mediated recognition and degradation of mRNAs lacking a termination codon. Science.

[9] Frischmeyer PA (2002). An mRNA surveillance mechanism that eliminates transcripts lacking termination codons. Science.

[10] Ito-Harashima S, Kuroha K, Tatematsu T, Inada T (2007). Translation of the poly(A) tail plays crucial roles in nonstop mRNA surveillance via translation repression and protein destabilization by proteasome in yeast. Genes Dev..

[11] Lu J, Deutsch C (2008). Electrostatics in the ribosomal tunnel modulate chain elongation rates. J. Mol. Biol..

[12] Tsuboi T (2012). Dom34: Hbs1 plays a general role in quality-control systems by dissociation of a stalled ribosome at the 3′ end of aberrant mRNA. Mol. Cell.

[13] Joazeiro CAP (2017). Ribosomal stalling during translation: Providing substrates for ribosome-associated protein quality control. Annu. Rev. Cell Dev. Biol..

[14] Garzia A (2017). The E3 ubiquitin ligase and RNA-binding protein ZNF598 orchestrates ribosome quality control of premature polyadenylated mRNAs. Nat. Commun..

[15] Guydosh NR, Green R (2017). Translation of poly(A) tails leads to precise mRNA cleavage. RNA.

[16] Barros GC (2021). Rqc1 and other yeast proteins containing highly positively charged sequences are not targets of the RQC complex. J. Biol. Chem..

[17] Filbeck S, Cerullo F, Pfeffer S, Joazeiro CAP (2022). Ribosome-associated quality-control mechanisms from bacteria to humans. Mol. Cell.

[18] Ikeuchi K (2019). Collided ribosomes form a unique structural interface to induce Hel2-driven quality control pathways. EMBO J..

[19] Meydan S, Guydosh NR (2021). A cellular handbook for collided ribosomes: Surveillance pathways and collision types. Curr. Genet..

[20] Simms CL, Yan LL, Zaher HS (2017). Ribosome collision is critical for quality control during no-go decay. Mol. Cell.

[21] Matsuo Y, Inada T (2021). The ribosome collision sensor Hel2 functions as preventive quality control in the secretory pathway. Cell Rep..

[22] Matsuo Y (2020). RQT complex dissociates ribosomes collided on endogenous RQC substrate SDD1. Nat. Struct. Mol. Biol..

[23] Sundaramoorthy E (2017). ZNF598 and RACK1 regulate mammalian ribosome-associated quality control function by mediating regulatory 40S ribosomal ubiquitylation. Mol. Cell.

[24] Juszkiewicz S, Speldewinde SH, Wan L, Svejstrup JQ, Hegde RS (2020). The ASC-1 complex disassembles collided ribosomes. Mol. Cell.

[25] Brandman O (2012). A ribosome-bound quality control complex triggers degradation of nascent peptides and signals translation stress. Cell.

[26] Defenouillère Q, Fromont-Racine M (2017). The ribosome-bound quality control complex: From aberrant peptide clearance to proteostasis maintenance. Curr. Genet..

[27] Winz ML, Peil L, Turowski TW, Rappsilber J, Tollervey D (2019). Molecular interactions between Hel2 and RNA supporting ribosome-associated quality control. Nat. Commun..

[28] Shen PS (2015). Rqc2p and 60S ribosomal subunits mediate mRNA-independent elongation of nascent chains. Science.

[29] Kostova KK (2017). CAT-tailing as a fail-safe mechanism for efficient degradation of stalled nascent polypeptides. Science.

[30] Kuroha K, Zinoviev A, Hellen CUT, Pestova TV (2018). Release of ubiquitinated and non-ubiquitinated nascent chains from stalled mammalian ribosomal complexes by ANKZF1 and Ptrh1. Mol. Cell.

[31] Osuna BA, Howard CJ, Subheksha KC, Frost A, Weinberg DE (2017). In vitro analysis of RQC activities provides insights into the mechanism and function of CAT tailing. Elife.

[32] Crabtree GR, Olson EN (2002). NFAT signaling: Choreographing the social lives of cells. Cell.

[33] Wang J (2021). The identification of a tumor infiltration CD8+ T-cell gene signature that can potentially improve the prognosis and prediction of immunization responses in papillary renal cell carcinoma. Front. Oncol..

[34] Cai Z (2006). hnulp1, a basic helix-loop-helix protein with a novel transcriptional repressive domain, inhibits transcriptional activity of serum response factor. Biochem. Biophys. Res. Commun..

[35] Zhang X (2020). Nulp1 alleviates cardiac hypertrophy by suppressing nfat3 transcriptional activity. J. Am. Heart Assoc..

[36] Chu J (2009). A mouse forward genetics screen identifies LISTERIN as an E3 ubiquitin ligase involved in neurodegeneration. Proc. Natl. Acad. Sci. USA.

[37] Choe YJ (2016). Failure of RQC machinery causes protein aggregation and proteotoxic stress. Nature.

[38] Udagawa T (2021). Failure to degrade CAT-tailed proteins disrupts neuronal morphogenesis and cell survival. Cell Rep..

[39] Martin PB (2020). NEMF mutations that impair ribosome-associated quality control are associated with neuromuscular disease. Nat. Commun..

[40] Stein KC, Morales-Polanco F, van der Lienden J, Rainbolt TK, Frydman J (2022). Ageing exacerbates ribosome pausing to disrupt cotranslational proteostasis. Nature.

[41] Sinha NK (2020). EDF1 coordinates cellular responses to ribosome collisions. Elife.

[42] Juszkiewicz S (2020). Ribosome collisions trigger cis-acting feedback inhibition of translation initiation. Elife.

[43] Morita M (2012). A novel 4EHP-GIGYF2 translational repressor complex is essential for mammalian development. Mol. Cell Biol..

[44] Park J, Park J, Lee J, Lim C (2021). The trinity of ribosome-associated quality control and stress signaling for proteostasis and neuronal physiology. BMB Rep..

[45] Weber R (2020). 4EHP and GIGYF1/2 Mediate translation-coupled messenger RNA decay. Cell Rep..

[46] Ikeuchi K, Inada T (2016). Ribosome-associated Asc1/RACK1 is required for endonucleolytic cleavage induced by stalled ribosome at the 3′ end of nonstop mRNA. Sci. Rep..

[47] Hickey KL (2020). GIGYF2 and 4EHP inhibit translation initiation of defective messenger RNAs to assist ribosome-associated quality control. Mol. Cell.

[48] Nielsen MH, Flygaard RK, Jenner LB (2017). Structural analysis of ribosomal RACK1 and its role in translational control. Cell Signal..

[49] Alagar Boopathy LR, Beadle E, Garcia-Bueno Rico A, Vera M (2023). Proteostasis regulation through ribosome quality control and no-go-decay. Wiley Interdiscip. Rev. RNA.

[50] Iyer KV, Müller M, Tittel LS, Winz ML (2023). Molecular highway patrol for ribosome collisions. ChemBioChem.

[51] Wang D (2019). A deep proteome and transcriptome abundance atlas of 29 healthy human tissues. Mol. Syst. Biol..

[52] Jiang L (2020). A quantitative proteome map of the human body. Cell.

[53] Agarwal V, Kelley D (2022). The genetic and biochemical determinants of mRNA degradation rates in mammals. Genome Biol..

[54] Chothani SP (2022). A high-resolution map of human RNA translation. Mol Cell.

[55] Mathieson T (2018). Systematic analysis of protein turnover in primary cells. Nat. Commun..

[56] Tsherniak A (2017). Defining a cancer dependency map. Cell.

[57] Collins RL (2022). A cross-disorder dosage sensitivity map of the human genome. Cell.

[58] Replogle JM (2022). Mapping information-rich genotype-phenotype landscapes with genome-scale Perturb-seq. Cell.

[59] Consortium, Gte (2020). The GTEx Consortium atlas of genetic regulatory effects across human tissues. Science.

[60] Consortium, T. M. (2018). Single-cell transcriptomics of 20 mouse organs creates a Tabula Muris. Nature.

[61] Vogel C, Marcotte EM (2012). Insights into the regulation of protein abundance from proteomic and transcriptomic analyses. Nat. Rev. Genet..

[62] Greenbaum D, Colangelo C, Williams K, Gerstein M (2003). Comparing protein abundance and mRNA expression levels on a genomic scale. Genome Biol..

[63] Maier T, Güell M, Serrano L (2009). Correlation of mRNA and protein in complex biological samples. FEBS Lett..

[64] Wolf AS, Grayhack EJ (2015). Asc1, homolog of human RACK1, prevents frameshifting in yeast by ribosomes stalled at CGA codon repeats. RNA.

[65] Matsuo Y (2017). Ubiquitination of stalled ribosome triggers ribosome-associated quality control. Nat. Commun..

[66] López-Otín C, Blasco MA, Partridge L, Serrano M, Kroemer G (2013). The hallmarks of aging. Cell.

[67] Chiti F, Dobson CM (2006). Protein misfolding, functional amyloid, and human disease. Annu. Rev. Biochem..

[68] Sitron CS, Brandman O (2020). Detection and degradation of stalled nascent chains via ribosome-associated quality control. Annu. Rev. Biochem..

