# Supplementary material for: Transcriptional profile of ribosome-associated quality control components and their associated phenotypes in mammalian cells
Source: Sci Rep. 2024 Jan 16;14:1439. doi: 10.1038/s41598-023-50811-z (PMC10792078; doi:10.1038/s41598-023-50811-z)
Supplement: Supplementary file 1 — Supplementary Information. [file 41598_2023_50811_MOESM1_ESM.pdf]

Supplementary files

Transcriptional profile of ribosome-associated quality control components and their associated phenotypes in mammalian cells

Otávio Augusto Leitão Dos Santos[1], Rodolfo L. Carneiro[1], Rodrigo D. Requião[2], Marcelo Ribeiro-Alves[3], Tatiana Domitrovic[4] and Fernando L. Palhano[1]*

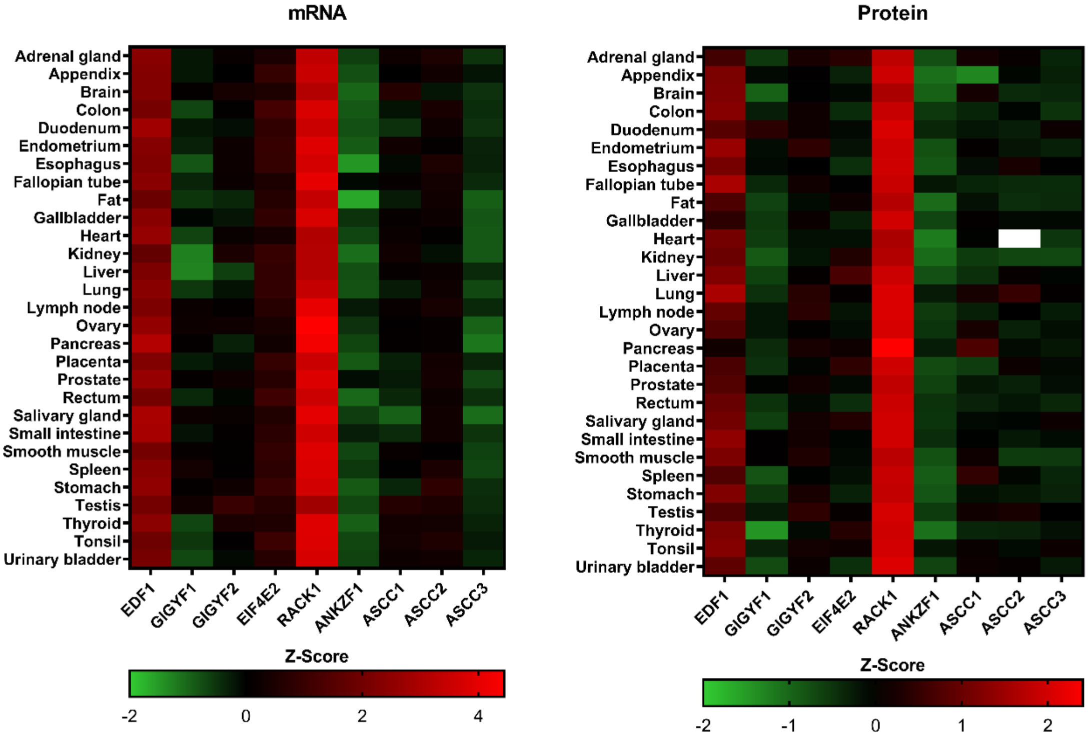

Supplementary Figure 1. Heatmap of the Z-scores distributions of the RQC-associated components calculated from the transcriptomes and proteomes of human tissue samples. Data obtained from D. Wang et al., 2019. White rectangles denote the absence of data. Each rectangle represents the expression of one of the RQC-associated genes against the average expression of the whole transcriptome/proteome in a particular tissue.

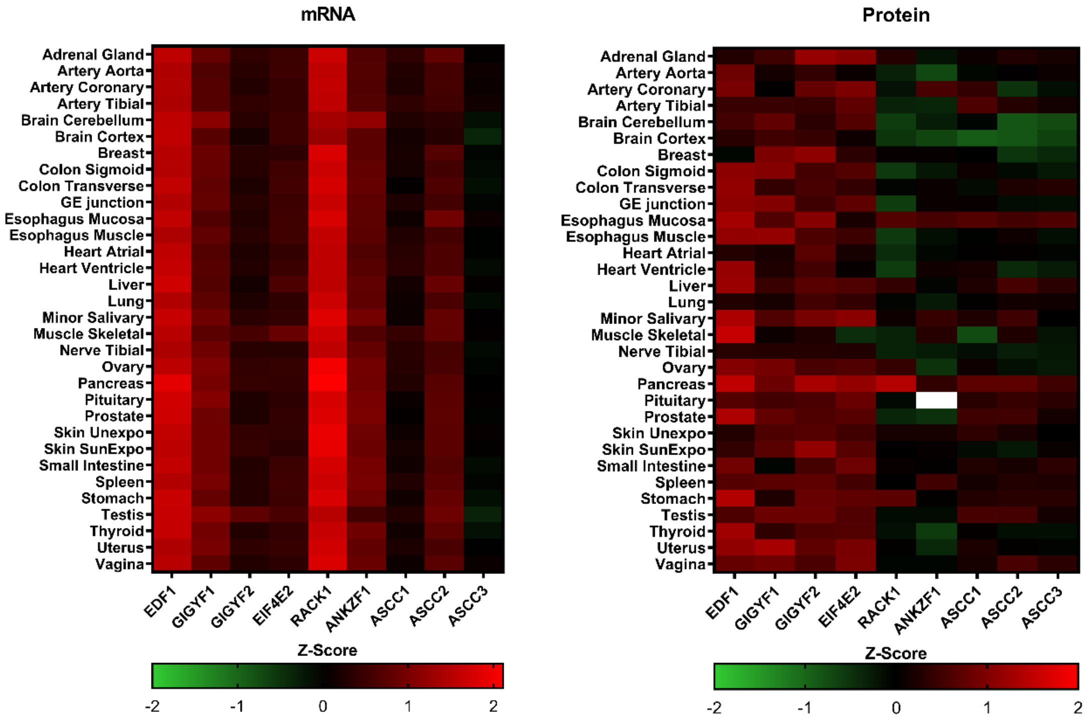

Supplementary Figure 2. Heatmap of the Z-Scores distributions of the RQC-associated components calculated from the transcriptome and proteome of 114 samples of 32 major organs from 14 different individuals, with replicates ranging from 1–22. Data obtained from Jiang et al., 2020. White rectangles denote the absence of data.

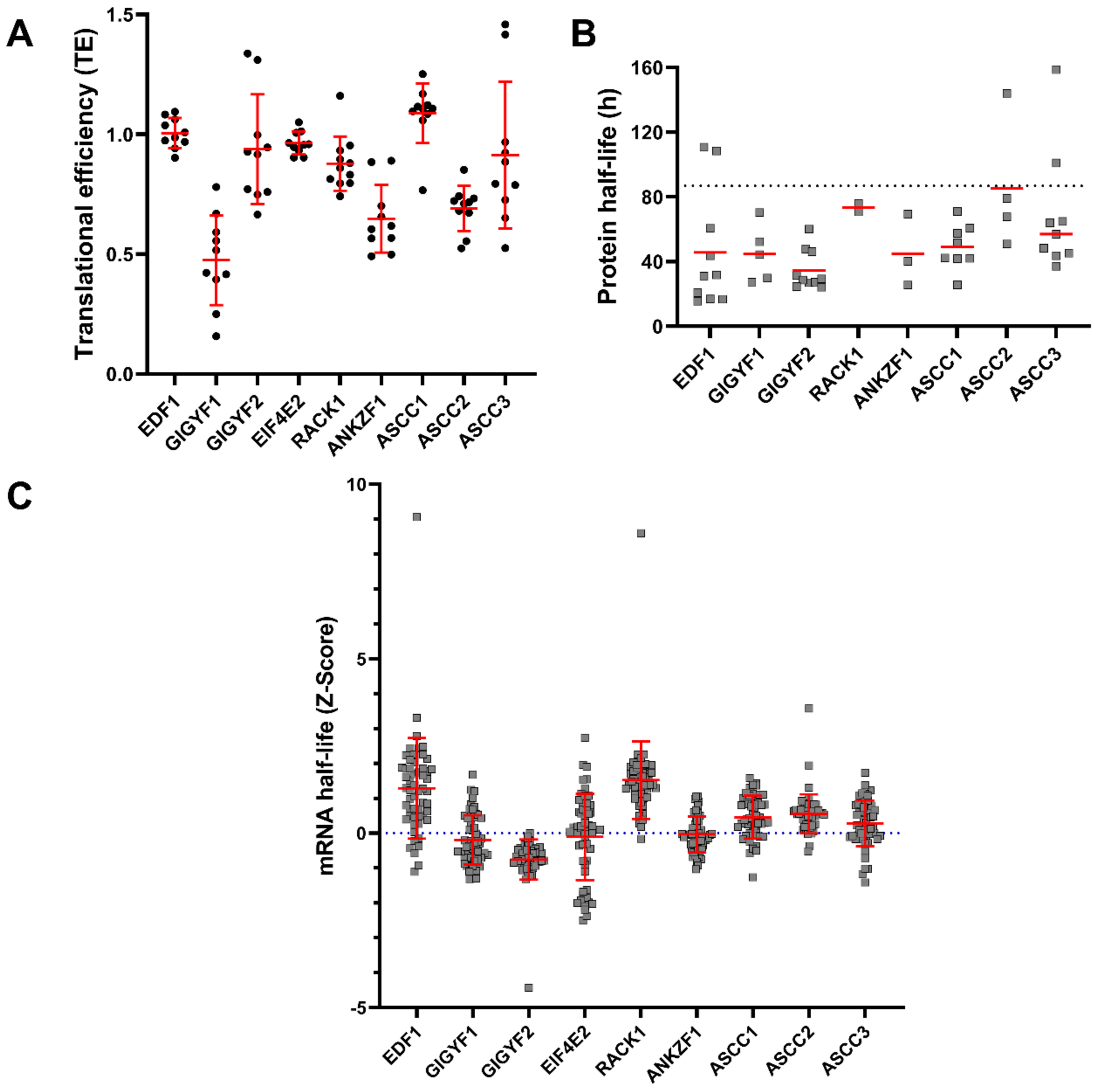

Supplementary Figure 3. Factors that affect the abundance of RQC-associated components. (A) Log_2 values of the mRNA translation efficiency of each transcript from six primary human cell types and five tissues. Data obtained from Chothani et al., 2022. (**B**) Protein half-lives for each RQC-associated components. The dotted line refers to the average half-life for all proteins considering all cell lines tested (86.95 hours). (**C**) Z-scores calculated for the half-lives of RQC-associated components transcripts in human cells. Data obtained from Agarwal V and Kelley D, 2022.2.

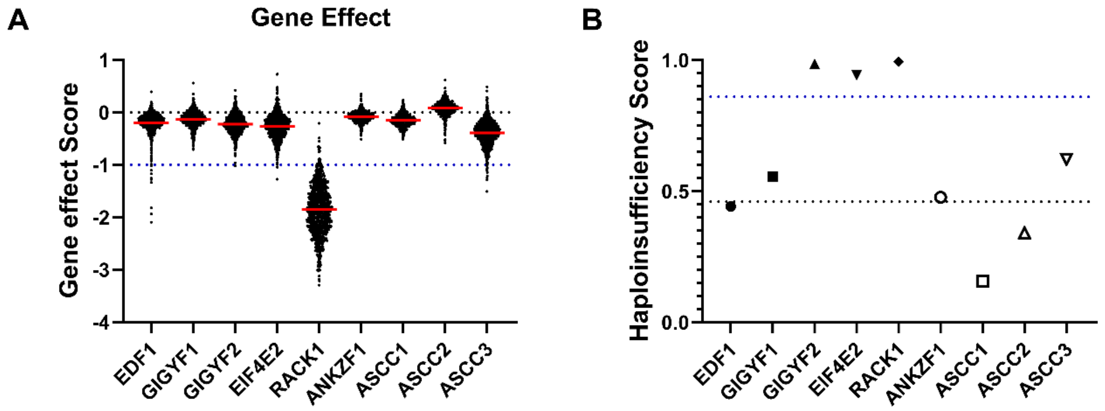

Supplementary Figure 4. The gene effect and haploinsufficiency score for RQC-associated components (A) Scores attributed to the impact of gene inactivation on growth rate in 1086 cancer cell lines for each RQC-associated component. If the gene inactivation caused no effect on cellular growth in a specific cell lineage, a score equal to zero **(dotted black line)** and a score of -1 **(blue dotted line)** meant that this pair led to cell death. Data obtained from Tsherniak et al., 2017. **(B)** Haploinsufficiency score for each RQC-associated component. Genes with values equal to or greater than 0.86 are considered haploinsufficient. Data obtained from Collins et al., 2022.

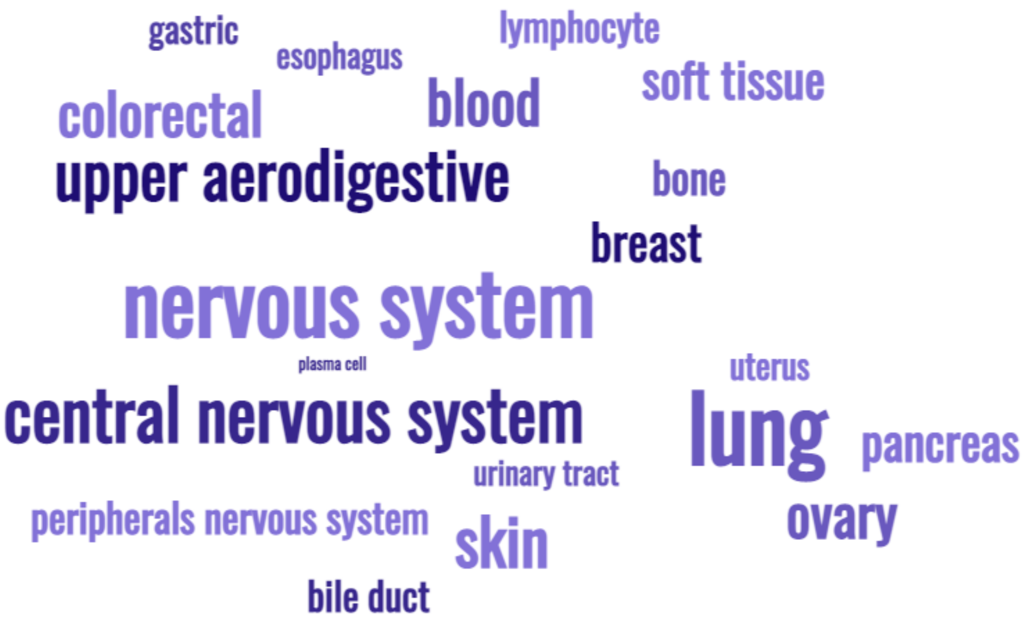

Supplementary Figure 5. Word cloud representation of the cellular lineages used to determine the impact of CRISPR-individual gene inactivation for LTN1, NEMF, and TCF25. Data obtained from Tsherniak et al., 2017.

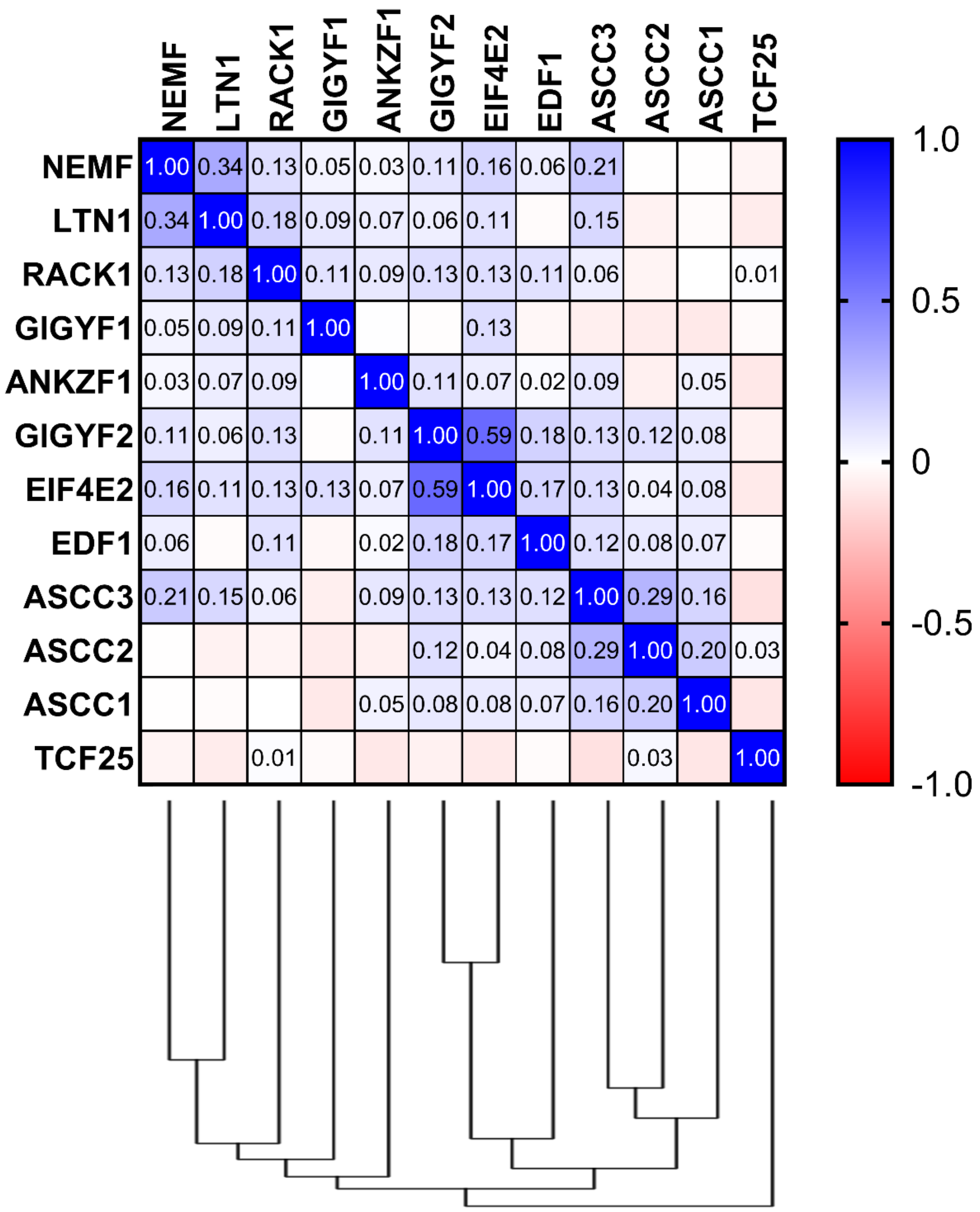

Supplementary Figure 6. Clustered correlation matrix of the silencing effect between RQC complex and RQC-associated components. The Person correlation among each pair was performed as in Figure 4B.

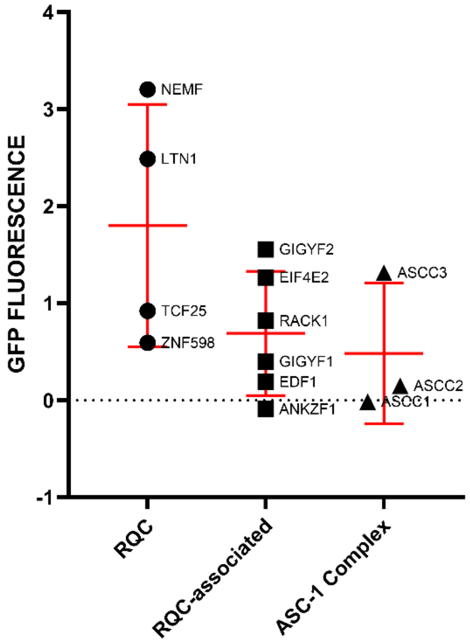

Supplementary Figure 7. Graph of GFP fluorescence for CRISPRi knockdowns of RQC complex, RQC-associated components, and ASC-1 complex genes.

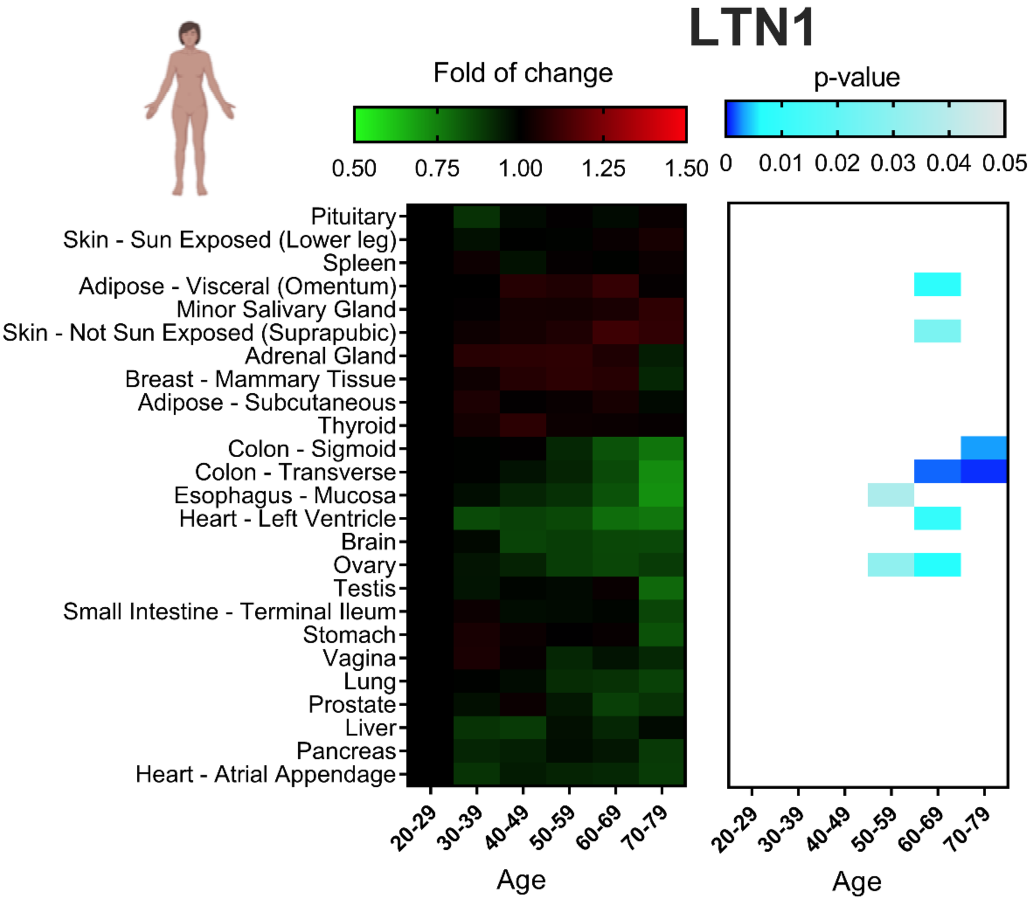

Supplementary Figure 8. Distribution of the Z-scores of the relative abundance of LTN1 mRNA in main human organs in different age clusters. Statistical significance was calculated using p-value and presented in a blue gradient for those samples that showed relevance. All data were normalized by the reference age value (20-29).

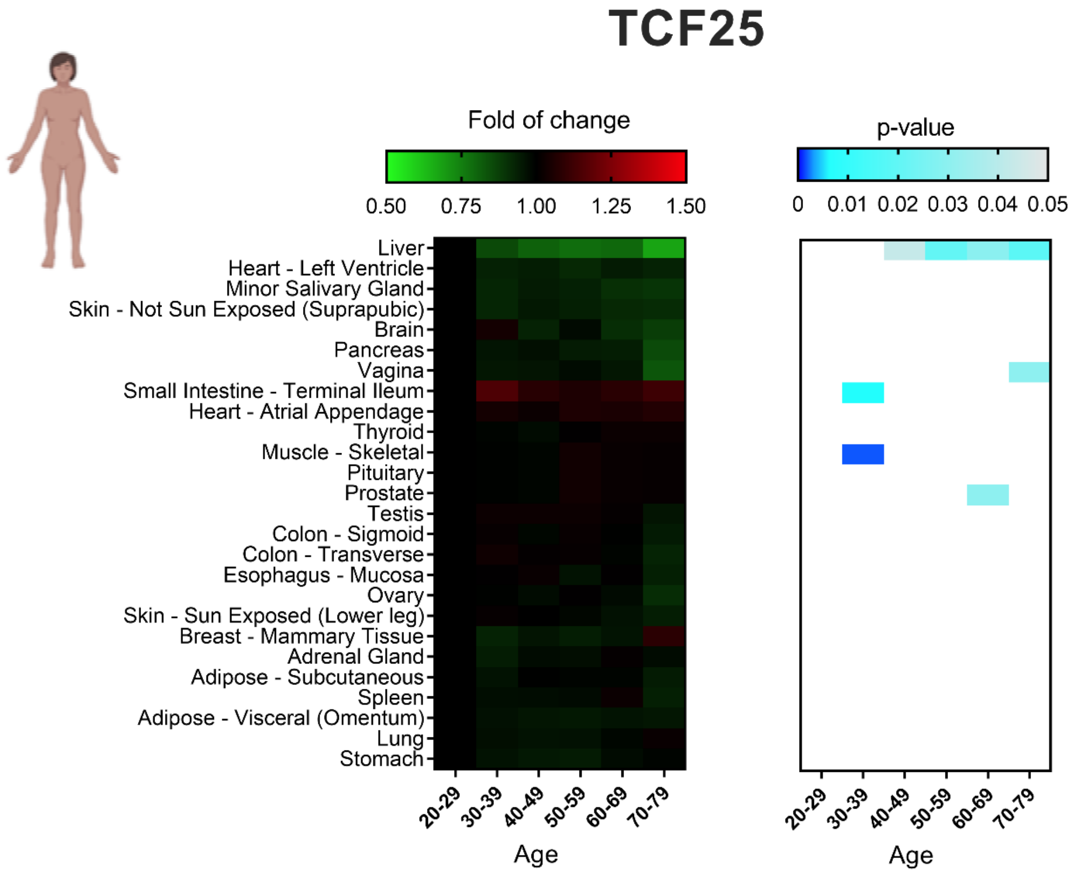

Supplementary Figure 9. Distribution of the Z-scores of the relative abundance of TCF25 mRNA in main human organs in different age clusters. Statistical significance was calculated using p-value and presented in a blue gradient for those samples that showed relevance. All data were normalized by the reference age value (20-29).

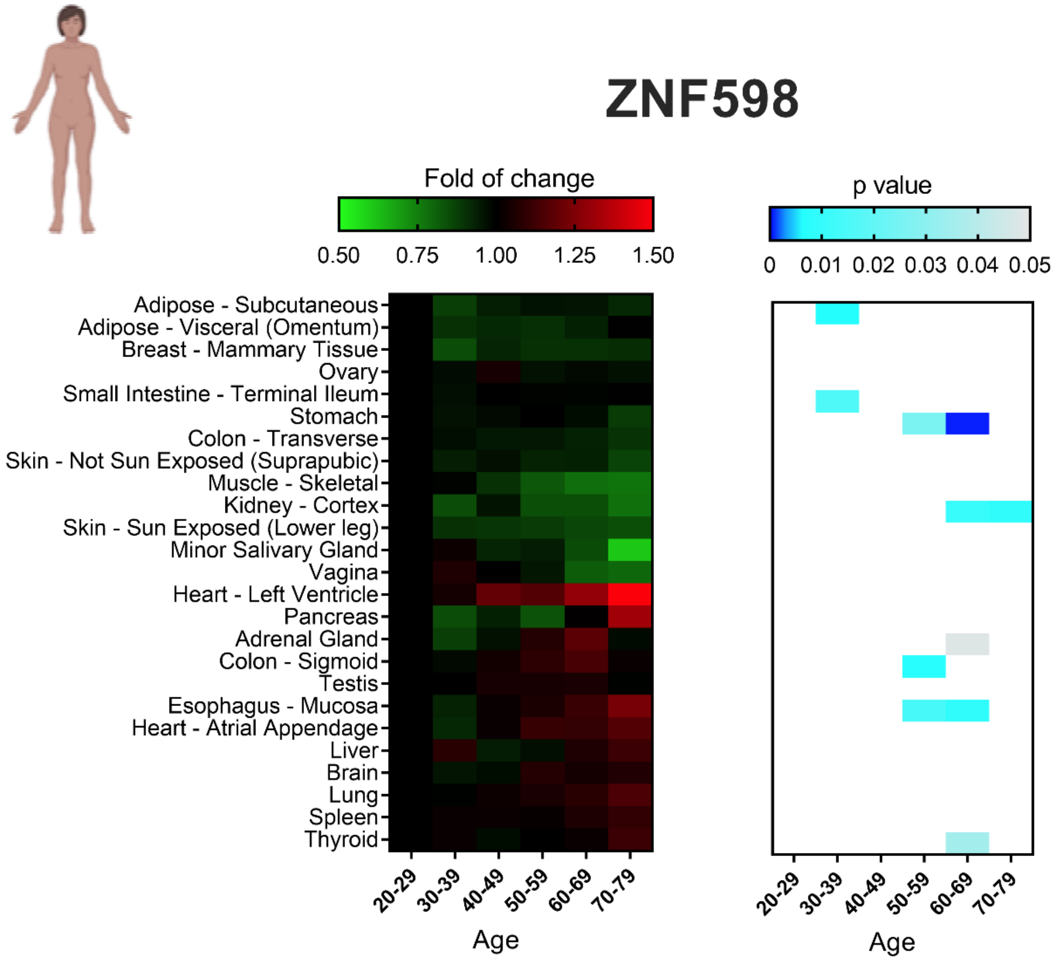

Supplementary Figure 10. Distribution of the Z-scores of the relative abundance of ZNF598 mRNA in main human organs in different age clusters. Statistical significance was calculated using p-value and presented in a blue gradient for those samples that showed relevance.

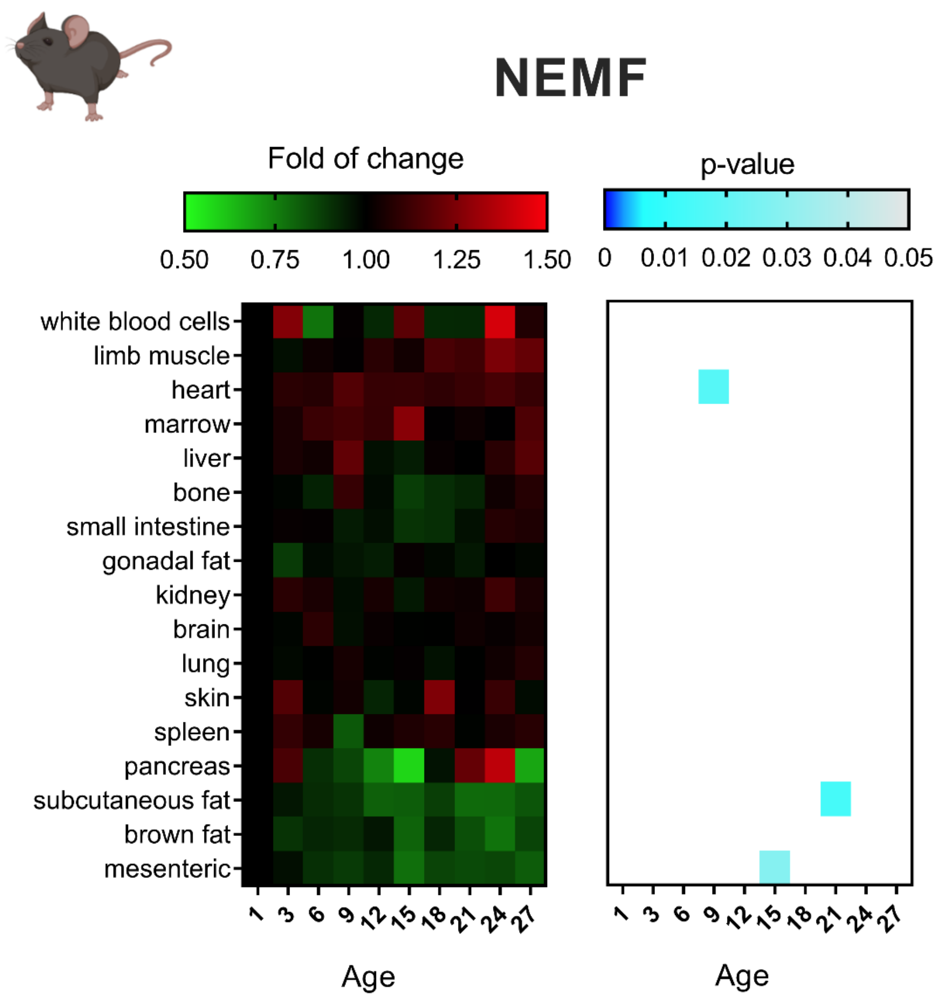

Supplementary Figure 11. Distribution of the Z-scores of the relative abundance of NEMF mRNA in main mouse organs in different age clusters. Statistical significance was calculated using p-value and presented in a blue gradient for those samples that showed relevance. All data were normalized by the reference age value (1 week).

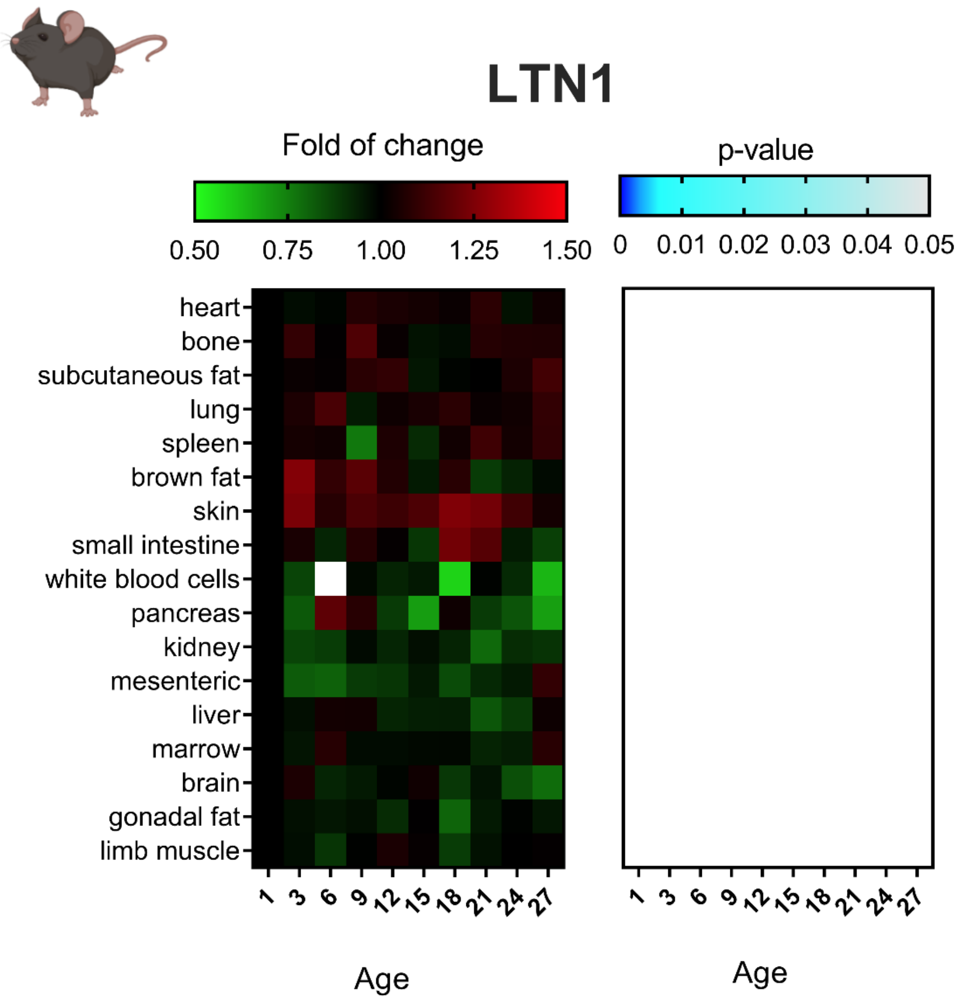

Supplementary Figure 12. Distribution of the Z-scores of the relative abundance of LTN1 mRNA in main mouse organs in different age clusters. Statistical significance was calculated using p-value and presented in a blue gradient for those samples that showed relevance. All data were normalized by the reference age value (1 week).

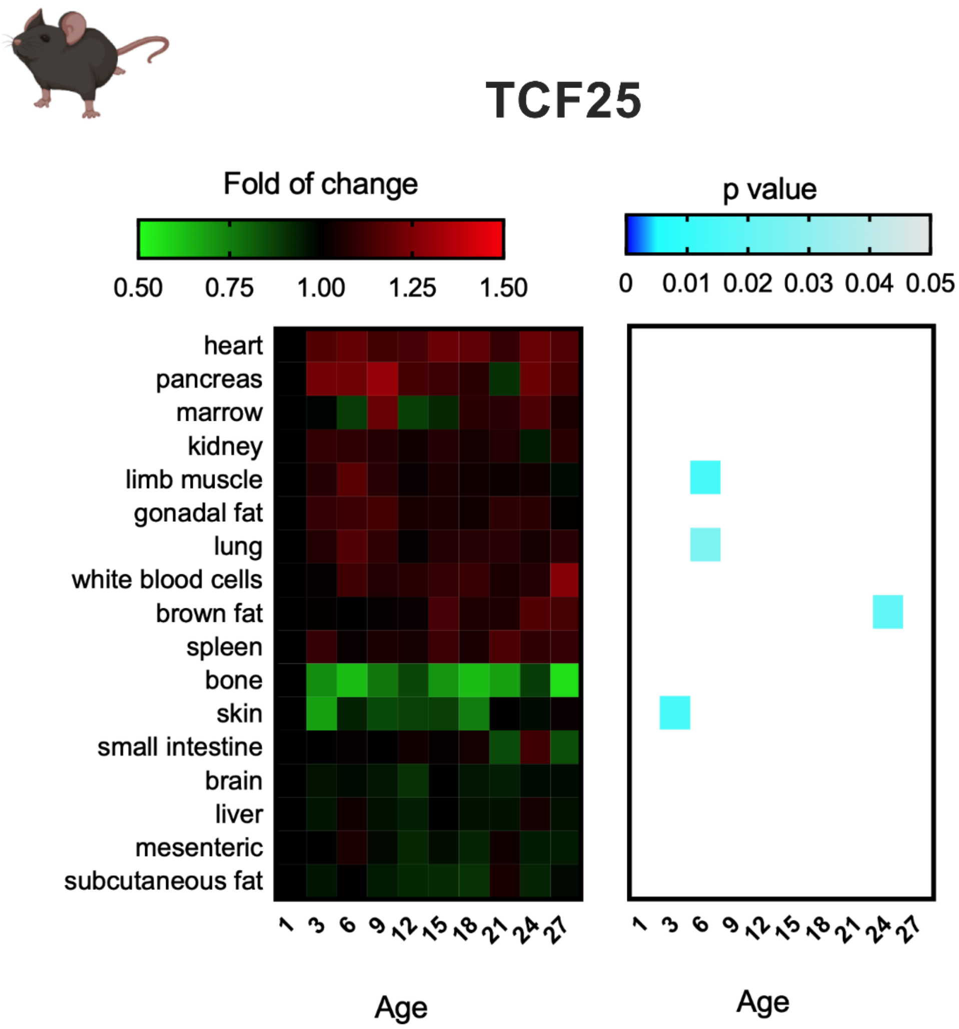

Supplementary Figure 13. Distribution of the Z-scores of the relative abundance of TCF25 mRNA in main mouse organs in different age clusters. Statistical significance was calculated using p-value and presented in a blue gradient for those samples that showed relevance. All data were normalized by the reference age value (1 week).

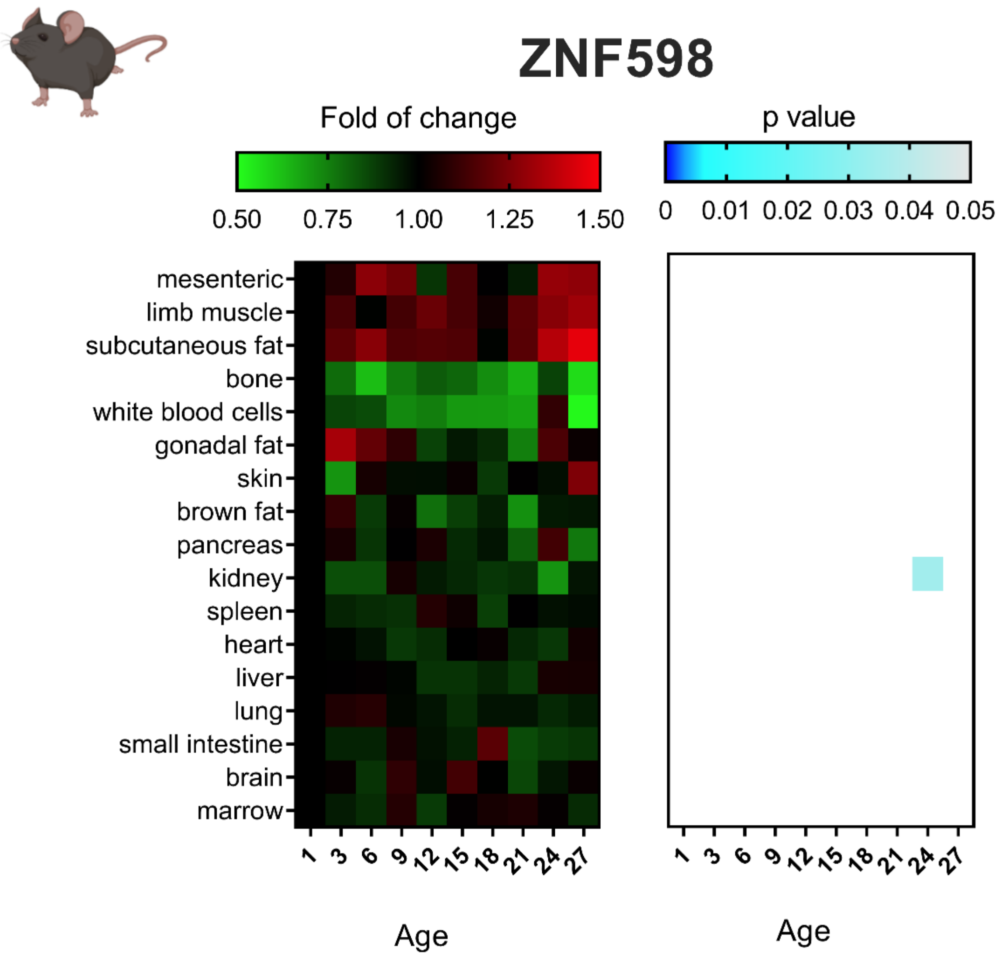

Supplementary Figure 14. Distribution of the Z-scores of the relative abundance of ZNF598 mRNA in main mouse organs in different age clusters. Statistical significance was calculated using p-value and presented in a blue gradient for those samples that showed relevance. All data were normalized by the reference age value (1 week).

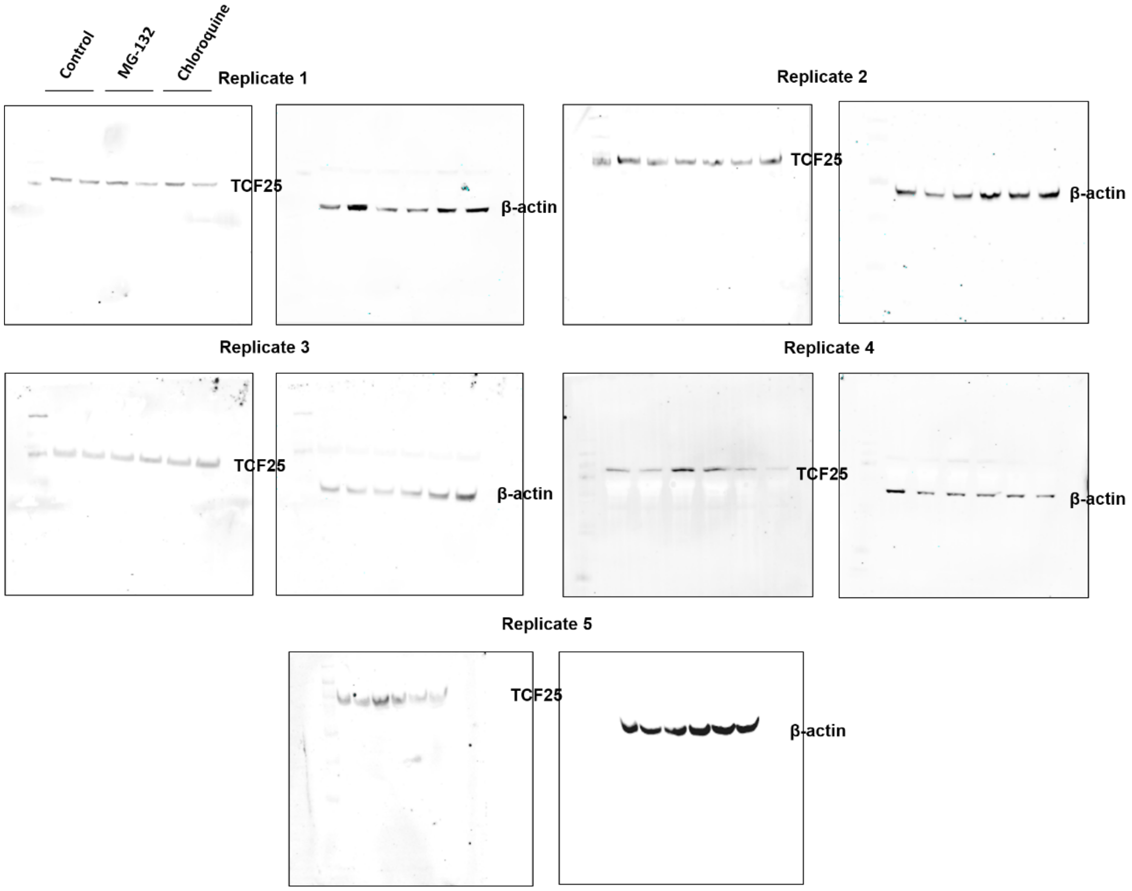

Supplementary Figure 15. Complete Western Blotting of different replicates used to quantify TCF25 protein levels during proteasome inhibition and autophagy in Figure 3D. Quantification was performed using ImageJ software. The lane sequence of all western blotting is similar to the first.

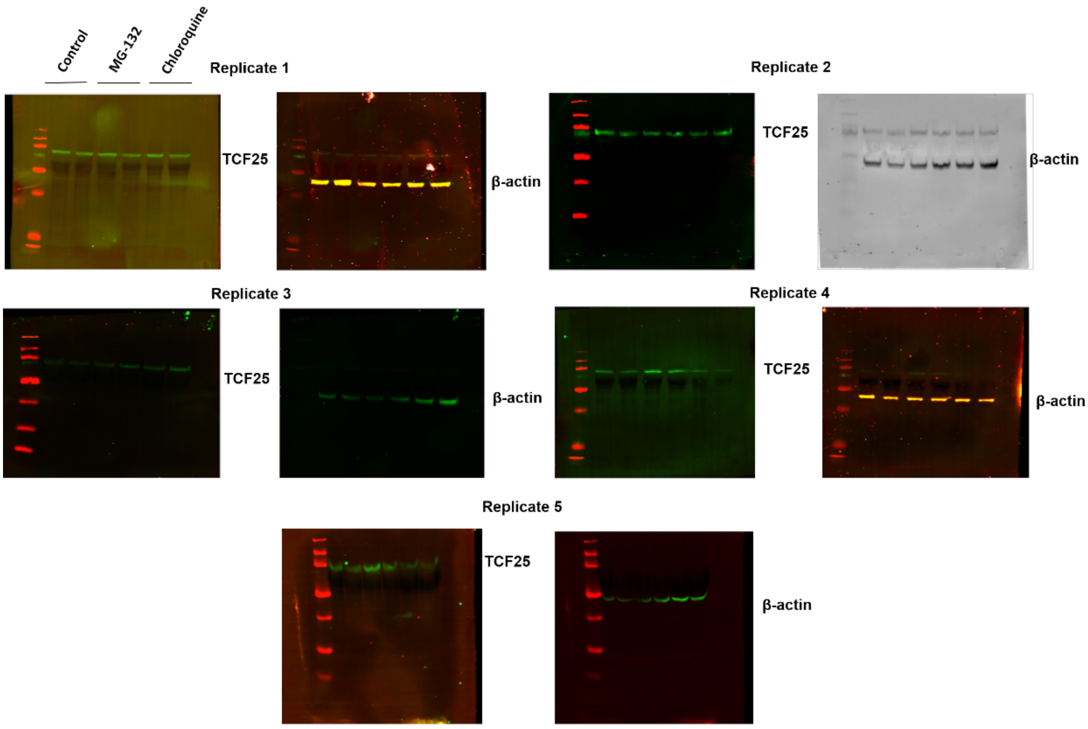

Supplementary Figure 16. Untreated Western Blotting of different replicates used to quantify TCF25 protein levels during proteasome inhibition and autophagy in Figure 3D. The lane sequence of all western blotting is similar to the first.

Supplementary Table 1: Gene function of the 7 genes with similar transcriptional profile with NEMF and LTN1 obtained from Ensembl.

Gene name	Ensembl ID	GO: Biological process
TMA16	ENSG00000198498	Ribosome biogenesis
SAPTA5L1	ENSG00000171763	Ribosome biogenesis
HERC1	ENSG00000103657	Autophagy<br>Brain development Protein ubiquitination Cerebellar Purkinje cell differentiation<br>Corpus callosum development<br>Neuron projection development<br>Regulation of catalytic activity<br>Neuromuscular process controlling balance
TEX10	ENSG00000136891	No available
LAS1L	ENSG00000001497	rRNA processing and maturation<br>Nucleic acid phosphodiester bond hydrolysis
DDX55	ENSG00000111364	No available
SURF6	ENSG00000148296	Ribosome biogenesis
